# Impacts of commodity prices and governance on the expansion of tropical agricultural frontiers

**DOI:** 10.1038/s41598-024-59446-0

**Published:** 2024-04-22

**Authors:** Javier Miranda, Wolfgang Britz, Jan Börner

**Affiliations:** 1 Institute for Food and Resource Economics, University of Bonn, Nussallee 21, 53115 Bonn, Germany; 2 Center for Development Research, University of Bonn, Genscherallee 3, 53113 Bonn, Germany

**Keywords:** Environmental economics, Environmental impact, Sustainability

## Abstract

Deforestation in the tropics remains a significant global challenge linked to carbon emissions and biodiversity loss. Agriculture, forestry, wildfires, and urbanization have been repeatedly identified as main drivers of tropical deforestation. Understanding the underlying mechanisms behind these direct causes is crucial to navigate the multiple tradeoffs between competing forest uses, such as food and biomass production (SDG 2), climate action (SDG 13), and life on land (SDG 15). This paper develops and implements a global-scale empirical approach to quantify two key factors affecting land use decisions at tropical forest frontiers: agricultural commodity prices and national governance. It relies on data covering the period 2004–2015 from multiple public sources, aggregated to countries and agro-ecological zones. Our analysis confirms the persistent influence of commodity prices on agricultural land expansion, especially in forest-abundant regions. Economic and environmental governance quality co-determines processes of expansion and contraction of agricultural land in the tropics, yet at much smaller magnitudes than other drivers. We derive land supply elasticities for direct use in standard economic impact assessment models and demonstrate that our results make a difference in a Computable General Equilibrium framework.

## Introduction

Many of the tradeoffs involved in achieving the UN Sustainable Development Goals materialize in the form of competing demands for land^1–3^. This has resulted in the continuous depletion of forest resources, with forest loss concentrating in the tropics during the past couple of decades. Tropical forest store vast amounts of carbon, regulate climate and water cycles, and host more than half of Earth’s species^4–10^. Agriculture, forestry, urbanization, and natural wildfires are known to be the main drivers of deforestation^11^, with agriculture alone accounting for more than 90% of recent forest losses^12^. Tradeoffs between competing forest uses, such as food and biomass production versus climate regulating functions and habitat provision, are most pronounced in tropical regions^13,14^. Strategies to effectively manage these tradeoffs at global scale require a better understanding of the mechanisms behind the drivers of deforestation and forest degradation^15^. This paper provides global scale evidence on two such mechanisms, namely commodity prices and national governance, in a coherent empirical framework that is compatible with state-of-the-art impact assessment models^16^.

The growing literature on the drivers of land use and land cover change has contributed substantially to our understanding of the mechanisms behind agricultural land expansion and deforestation at national and regional scales^17,18^. However, only few studies have so far produced global-scale evidence (e.g. Berman et al*.*^19^) that could be used to inform model-based policy impact assessments as a basis for the design of a coherent global land governance framework. Moreover, global economic models^20^, build on a mechanistic understanding of agricultural systems and their interactions with the general economy. If properly parameterized, such models allow us to assess the potential impacts of alternative global and national policy regimes in scenario analyses^21^. This includes impacts on sustainability dimensions, such as food security, climate change mitigation, and biodiversity conservation^22,23^. An important challenge is to improve these models towards realistically representing spatial and contextual heterogeneity in the response of forest and agricultural land uses to market and policy incentives especially in data-scarce regions, such as the global tropics^16,24^.

A key parameter that characterizes the supply of new agricultural land, for example, through deforestation, is the so-called land supply elasticity^25,26^. Put simply, the land supply elasticity describes, for example, how much forest land is converted to agricultural uses in response to an increase in agricultural commodity prices. The higher the elasticity, the larger the expansion of agricultural land for a given price increase. Variations in land supply elasticities across countries could be due to differences in available agricultural technologies, government policies, or the functioning of land markets. Especially in tropical forest regions, agricultural expansion often takes place at the costs of forests. This is why reliable information on the size and variation of land supply elasticity is valuable information for applied research and policy makers.

Various studies have estimated land supply elasticities at national and regional scales using predictors that reflect land rents. For example, Barr et al.^27^ used agricultural sector data from Brazil and the US to investigate the effect of biofuel policies on cropland expansion. Villoria^28^ studied the effect of total factor productivity as a driver of cropland expansion in different regions worldwide. In a cross-sectional analysis, Villoria and Liu^25^ used gridded information to make spatially explicit calculations of the effect of accessibility on cropland expansion in the American continent. Liu and Villoria^29^ used a similar approach but Sub-Saharan Africa. Tabeau et al.^26^ conducted a literature review of different calculations of land supply elasticities to provide information for global economic models, assessing the European Union’s economy on the world stage.

This paper builds on these prior contributions with the aim to provide global-scale quantitative evidence on two antagonistic key determinants of agricultural land expansion (henceforth land supply) in the tropics, namely agricultural commodity prices and alternative dimensions of national governance^30,31^. We used remotely-sensed land cover information to identify agricultural land in different countries and agro-ecological zones (AEZs). We included pasture areas in agricultural land instead of only cropland as they are an important source of land use change in agriculture-forest frontiers^32^. Many studies rely on the Food and Agriculture Organization’s (FAO) survey data, which enable the structuring of information for a wide range of countries for empirical analysis. However, these data can underestimate the conversion of natural land for agricultural use as they are based on self-reported information. The recent explosion in the availability of satellite-based spatially explicit information on land use and land cover change and indicators of drivers thereof allows us to estimate land supply elasticities across the global tropics. We also used panel data structured at the subnational level to run our empirical analysis. Further, we expanded the analysis to tropical zones worldwide using a single framework. We focus on these regions as there has been land use expansion from agricultural activities in these regions^11,33–35^.

We specifically test for the effect of governance on land supply, because both conventional and environmental governance were shown to affect the availability and costs of access to land in geographically heterogeneous ways^36–38^. For example, rigorous environmental governance can artificially increase land scarcity and change local land and technology use decisions^39^. Governance can also have effects on surrounding jurisdictions. For instance, effective conservation policies can lead to spillover effects, which can have both positive and negative land use change impacts^40^. Moreover, different types of governance have different effects on land use. A study on South America revealed that the type of governance determines the direction of the underlying relationship^41^. Therefore, we tested the effect of conventional and environmental governance indicators. This included indicators of corruption, voice and accountability (V&Acc), and rule of law (RoL) as proxies for conventional governance as well as national terrestrial biome protection to represent environmental governance.

Utilizing the definition of the elasticity of land supply, which denotes the percentage change in agricultural land after a 1% increase in its rentability for agricultural production^25^, we laid the groundwork for our analytical framework. We then estimated a correlated random effects model suitable for fractional response variables, such as the share of agricultural land in the total land endowment, with panel data. Our results are compatible with prior work at national and regional scales. We found that an increment in commodity prices is positively associated with increments in the share of land allocated to agricultural use. We did not find a systematic positive impact of conventional governance on tropical forest conversion, but consistent evidence for a (small) negative relationship between environmental governance effectiveness and agricultural land expansion. Regarding subnational level land supply elasticities for each mesoregion, we found that an increase in agricultural commodity prices has a stronger effect in areas with large private forest reserves, such as the Amazon region in South America, the Congo Basin in Central Africa, and forestlands in Southeast Asia (particularly Indonesia). The mean value of the elasticities calculated across different specifications for a 1% change in the agricultural commodity price index is 0.1% of land supply (with a minimum value of 0.01% and a maximum of 0.34%).

## Results

### Forest and agricultural land

**Figure 1:**
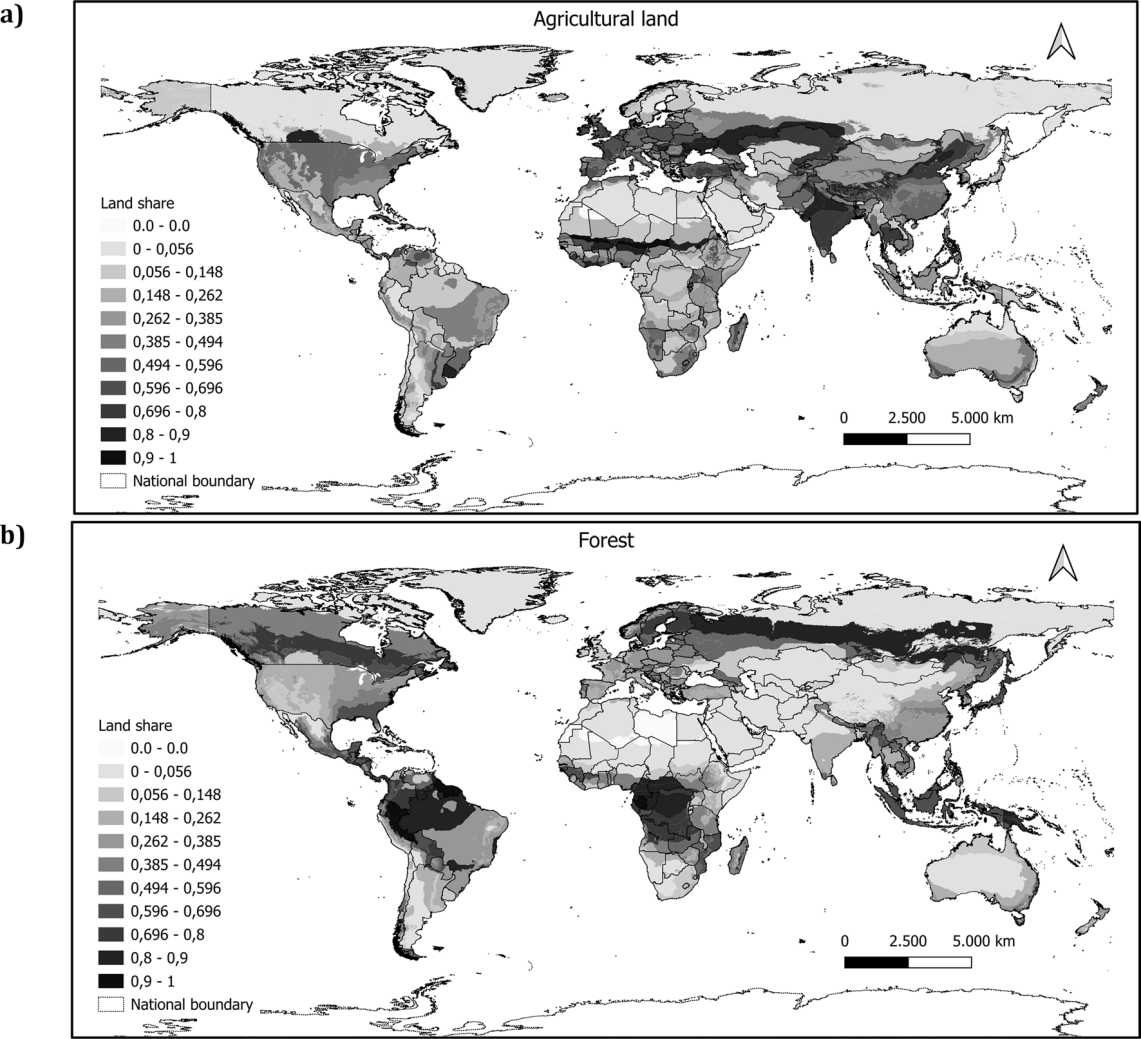
Land shares. (**a**) and (**b**) represent shares of land use endowment within each AEZ-country observation in 2015.

Figure 1a depicts the share of agricultural land to the total land endowment in each mesoregion, i.e., subnational agro-ecological zone (AEZ)‒country observations, in 2015. Temperate AEZs, i.e., zones 7–12 (see Supplementary Fig. S1), boast a high concentration of regions with an agricultural land share above 0.5. Areas in Central and Eastern Europe, the West of North America, and Asia primarily hold higher shares of their land for agricultural use. These patterns leave relatively little room to expand land supply for agriculture in these areas. In the tropical zones (AEZs 1–6), there are areas whose share ranges from 50 to 100%, such as India or the south of the Sahel region. However, this spatial pattern seems sparse and with less extension as compared with temperate zones. Figure 1b depicts forest shares per AEZ-country region. Here the pattern from the agricultural share is reversed. We find that besides regions with a high land endowment (e.g., those in Russia or Canada), the share of forest cover is relatively smaller compared with agricultural land shares in temperate zones (darker gray hues depict a higher share). Larger forest land shares are concentrated in the tropical zones, e.g., the Amazon, the Congolese, and the Indonesian rainforests. As previous analyses have pointed out, if the economic conditions allow for positive land rent expectations, agricultural land is likely to expand especially in forest abundant world regions.

As our definition of land supply implies a change in land use and forest is in competition with agricultural production for land, the remainder of this section focuses on subnational regions in the tropical zones.

**Figure 2:**
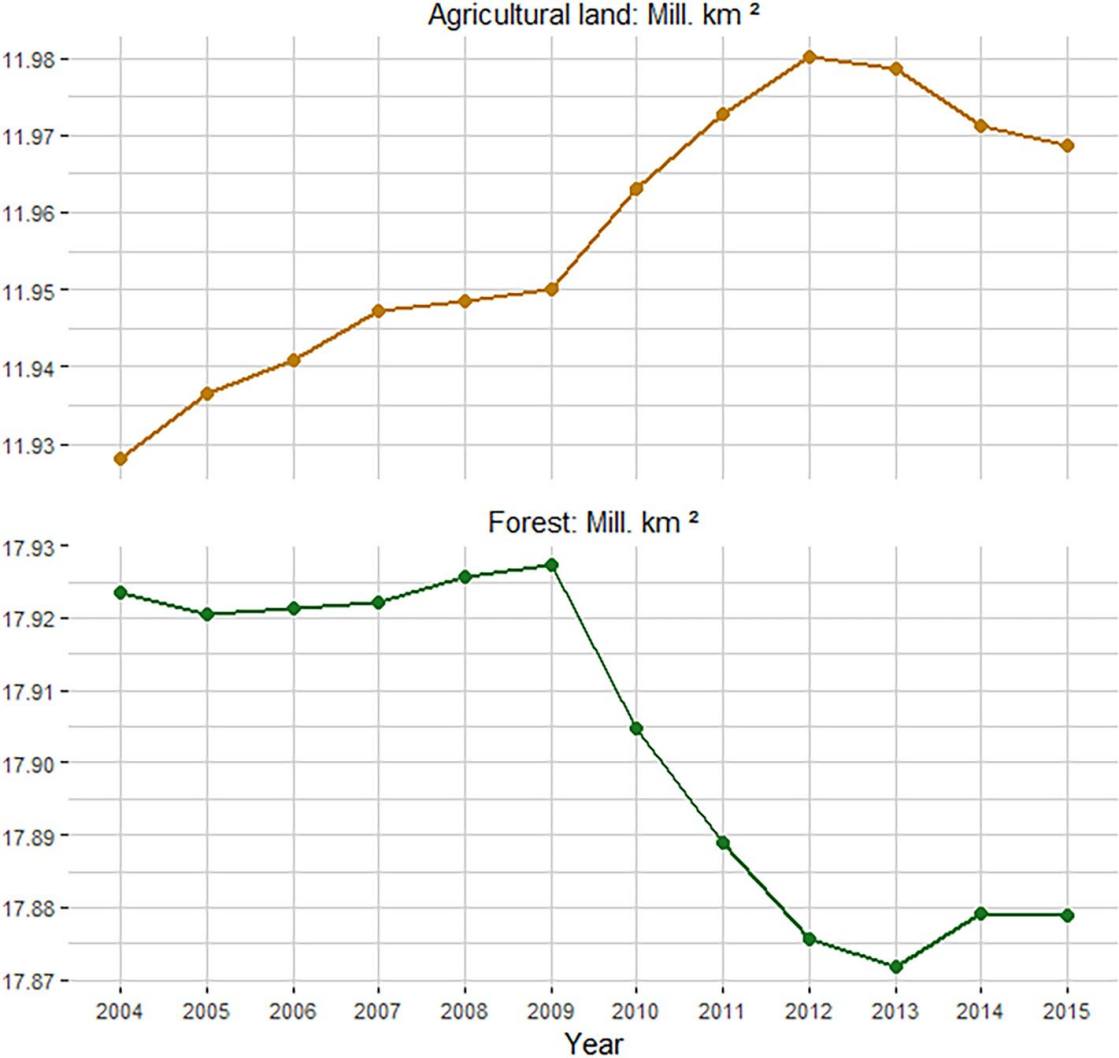
Forest and agricultural land in the tropics. Each line depicts the total size of land in each use within the tropical AEZs analyzed in the study.

The upper and lower panels of Fig. 2 depict the amount of land allocated to agricultural use and forest in the tropics from 2004 to 2015, respectively. Change in the levels of these two land use indicators can be divided into two periods. First, from 2005 to 2009, agricultural land and forest increased simultaneously, suggesting less competition between these land uses. Agricultural land increased by 0.11%, while forest areas in the tropics recovered 0.04% of the territory in the same period. This development may be related to some of the effects of conservation policies on reducing forest loss in Brazil in the mid-2000s^42–44^. However, the effects of conservation policies were fading away in the second decade of the 21st Century due to a continuous weakening in environmental governance in the South American country since 2012^45^. Second, from 2009 to 2015, the annual allocation of these two land uses moved in opposite directions, reinforcing the idea of competition in our sample^11,46^. For instance, from 2009 to 2012, agricultural land expanded by 0.25%, while forests lost 0.28% of their territory. Finally, the total change from 2004 to 2015 was 0.33% and −0.24% for agricultural land and forests, respectively.

### Drivers of land supply and forest loss

**Figure 3:**
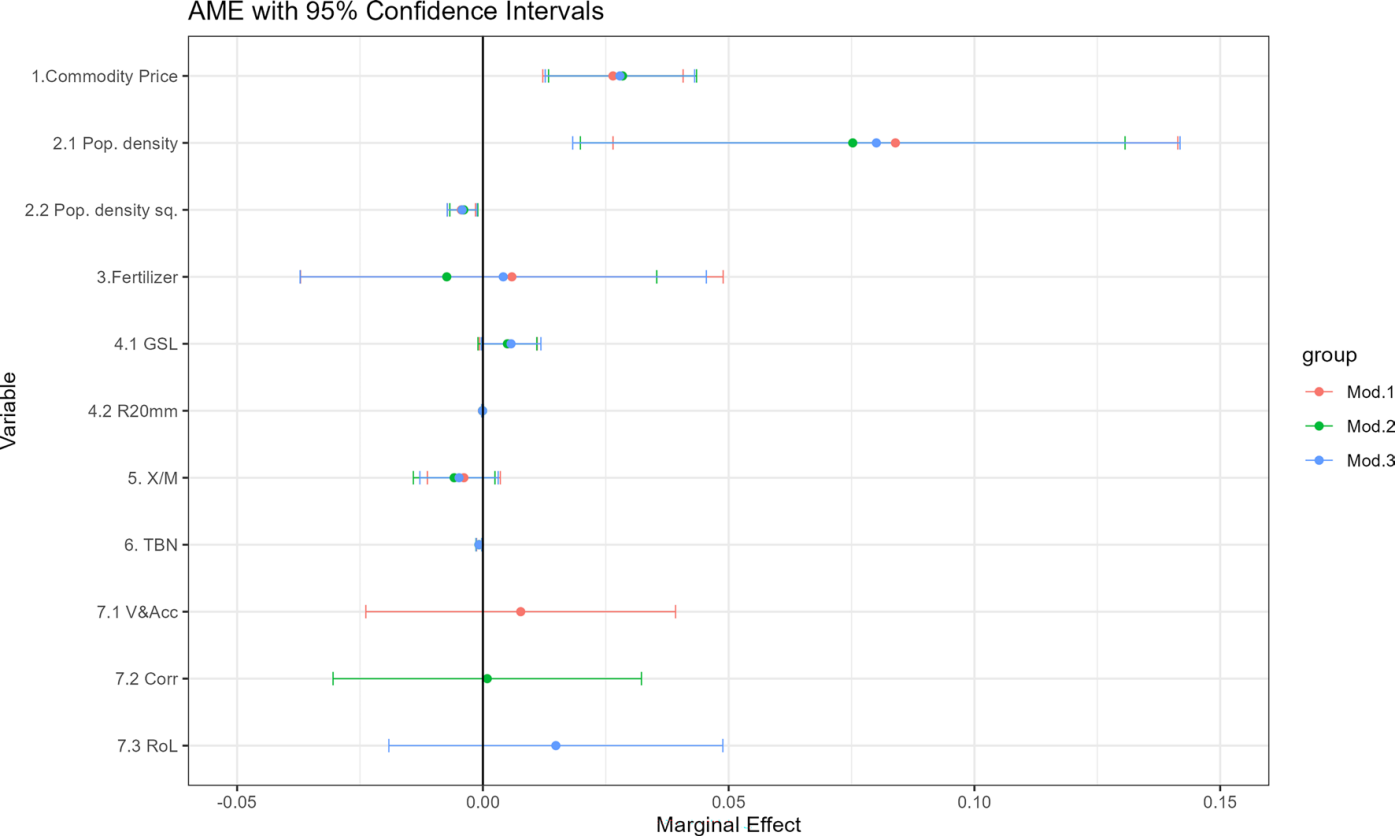
Average marginal effects. The figure depicts the average marginal effects for each of our covariates tested as drivers of land supply (see Material and methods for variable labels). Models (1) to (3) represent the different specifications using different conventional governance indicators, which are V&Acc (red), corruption (green), and RoL (blue).

We estimated nine models for the three governance and fertilizer use indicators (see “Materials and methods”). We only reported the results of one of the fertilizer use proxies (i.e., P205) because the main results did not change across specifications. Therefore, the specifications reported are similar, except for the variable representing conventional governance. Other results are available upon request. We did not include all the governance indicators in one estimation as they are highly correlated (see Supplementary Fig. S2). The econometric results of the fractional response models are presented in Supplementary Table S4. The scaled coefficients presented in the table only give information on the quality of the relationship between each covariate and our dependent variable—agricultural land share^47,48^. Therefore, we perform a normal transformation of the linear relationships to obtain the average marginal effects for tropical AEZs. The results are reported in Fig. 3, where points represent the estimated average marginal value and the length of the lines denotes confidence intervals at the 95% level. The numbering (Mod.1) to (Mod.3) denotes alternative model specifications, depending on the conventional governance indicator used, that is, V&Acc (red), corruption (green), and RoL (blue).

We found that the commodity price index is positively related to land supply, with an average marginal effect ranging from 0.026% to 0.028% across the different specifications. We used this result to calculate individual elasticities for each AEZ-country unit, which are presented in the next section. Regarding the other socioeconomic indicators, we found that the effect of population density is positively correlated with land supply and negatively correlated in its quadratic form. This suggests that although increases in population density pushed for land expansion, as the population continued to increase, it eventually reached an inflection point. The value-added ratio of agricultural exports to imports reveals a negative relation with land supply, although it is not statistically significant across the different specifications. Based on the available information, we could not find systematic evidence that when products are more costly in a country compared with international markets, the demand for land supply reduces. Moreover, we did not find a systematic relationship between the fertilizer indicator and land supply. This can be due to the low disaggregation in the fertilizer information that we used, which can blur its effect on land supply. Similarly, our bundle of indicators to account for the biophysical component of land rents indicate a small magnitude of scaled coefficients, where only the effect of the growing season period is statistically significant across specifications. The aggregation of these variables might lose some of the richness that spatial variation at the pixel level might capture. Improvements in conventional governance across all specifications and indicators have a positive relationship with increments in land supply, but this relationship is not statistically significant. The coefficients related to terrestrial biome protection suggest a negative relationship with land supply, though the average marginal effect is very small.

### Land supply at subnational level

**Figure 4:**
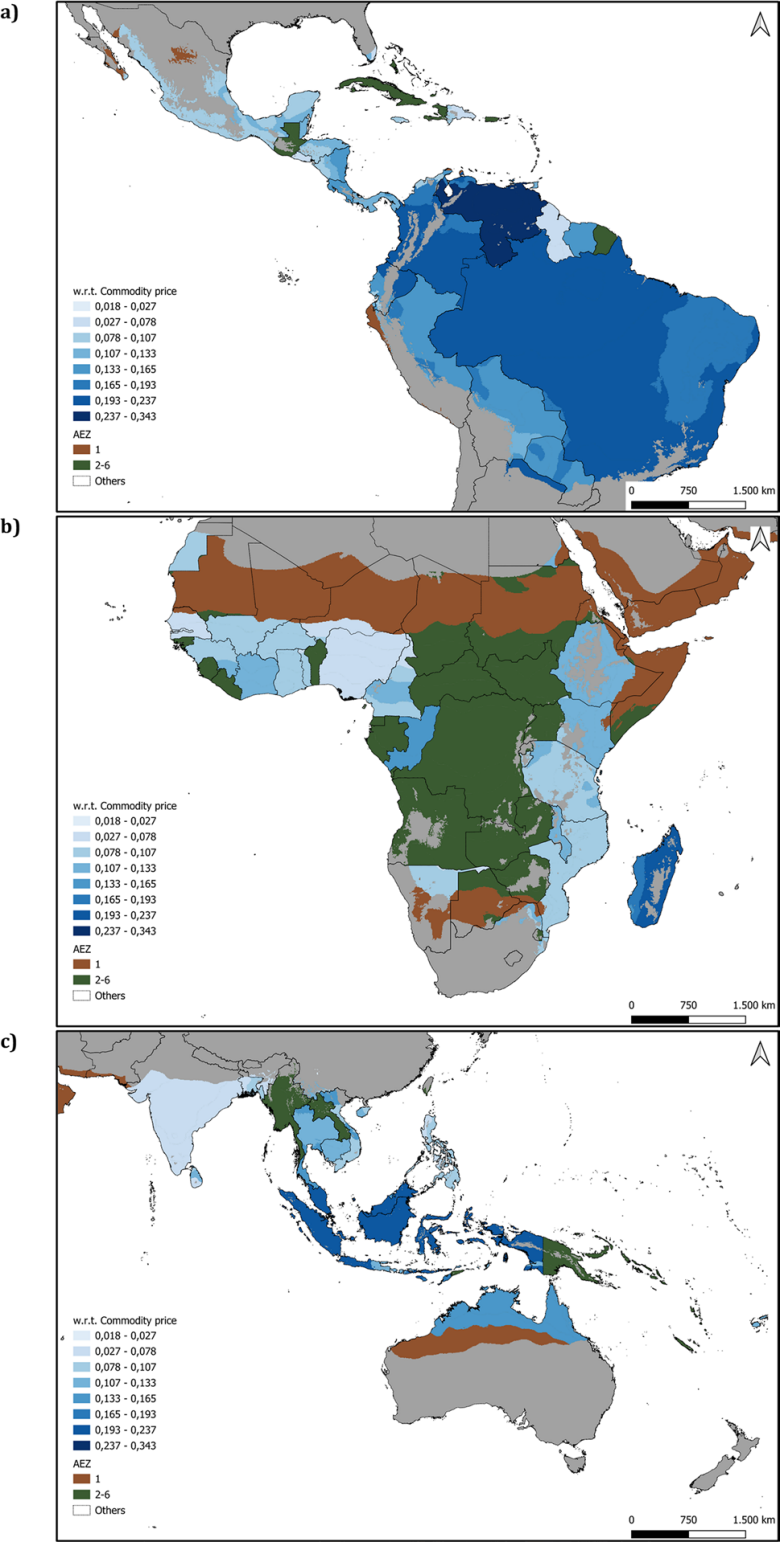
Subnational level land supply elasticities in the tropics. The subnational level elasticities depicted in (**a**), (**b**), and (**c**) are calculated using the coefficients estimated in column (3) of Supplementary Table S4. Each subnational mesoregion represents an AEZ-country observation. The shades of blue represent land supply elasticities to changes in the agricultural commodity price index during the research period. Green areas are tropical mesoregions for which we did not have information to test our model implementation. Brown areas are mesoregions in desert areas not considered in our estimation. Greater sensitivity (i.e., higher elasticities in darker hues) is located in areas where the extensive margin of agricultural production can be activated due to relatively abundant land resources, e.g., Brazil and Indonesia. The tabular format of the elasticities is presented in Supplementary Table S5.

We used the results in column (3) in Supplementary Table S4 to calculate individual land supply elasticities, i.e., specific to each unit of observation, as explained in the “Materials and methods”. We presented the results of this analysis in a spatial format in Fig. 4 (the table is presented in Supplementary Table S5 with the subnational elasticities calculated for all specifications), and the range of individual elasticities is from 0.018 to 0.343. It is important to clarify that the land supply elasticities depicted are measures of the sensitivity of agricultural land expansion to agricultural prices. They measure the percentage change in land supply expansion when there is a 1% change in prices. Therefore, we expect a different agricultural area expansion (in absolute terms) depending on the AEZ-country observation. For instance, the AEZ-5 in Brazil and Madagascar have a similar elasticity of approximately 0.20 for some of our specifications. This represents an expansion (compare to 2015 levels) of nearly 70 km^2^ in Madagascar compared with approximately 2577 km^2^ in Brazil per percentage increments in average yearly prices.

We found higher mean (0.14) and maximum (0.33) values in the AEZ 6, which comprises rainforests such as the Amazon rainforest, the Congolian rainforest, and those in Indonesia. Our estimations suggest a higher likelihood of agricultural land expansion in areas with vast forest endowments. This is not surprising as these areas also host business-oriented agricultural production linked to deforestation risks.

In contrast, lower values were found in highly populated areas with relatively little land available to expand agriculture, such as some mesoregions in China, Bangladesh, India, or Nigeria (with no more than 0.06 elasticity magnitudes). Small values were also found in areas with a less developed agricultural sector, such as Guyana, Jamaica, and the Dominican Republic.

In Africa, land use change from forest to agriculture happens more prominently due to small-scale shifting cultivation^11^. Unfortunately, we do not have sufficient information on all covariates of the complete model to calculate individual elasticities for important forested countries located in the Congo Basin, such as the Democratic Republic of the Congo, Angola, and Zambia (depicted with green fill in Fig. 4). However, we measured elasticities for other countries within this basin, such as the Republic of Congo (0.159 and 0.163 in AEZ 5 and 6, respectively) and Tanzania (from 0.099 to 0.128 across AEZs 3 to 6) in Central Africa. The country with the highest individual elasticities in Africa is Madagascar (from 0.149 to 0.216), which has been subject to high levels of deforestation in the past two decades.

The calculated individual elasticities suggest different flexibility of agricultural systems across the tropics. We found smaller elasticity values across Africa in comparison with South America or Southeast Asia. As agribusiness requires more capital, it can only be more flexible to changes in factors of production if there are more investments in fertilizer use or land use conversion. In Africa, agricultural systems are mostly characterized by small-scale production, while those in South America or Southeast Asia are large-scale export-oriented systems. Our results reveal this pattern across all mesoregions analyzed (see Fig. S3-S4 for a comparison between observed and estimated agricultural land expansion).

### Applying the estimated elasticities in CGE analysis

To illustrate how the estimated elasticities can inform model-based impact assessments, we conduct a comparative-static Computable General Equilibrium (CGE) analysis that explores impacts of a preference shift away from animal-based food towards a more vegetarian diet at global scale (see “Materials and methods”).

Previous analysis with a similar model layout used rather moderate land supply elasticities in the range of 0.05, calibrated to recover crop land expansion trends from another study^49^. We substitute these default values with our estimated elasticities where available and keep the default values for all non-tropical AEZ-country units.

The simulated demand changes for meat follow closely our preference shifts for the aggregate household. As meat is not only feeding into household demand, but also into intermediate demand, global and regional output changes are less noticeable. Both changes in demand and outputs are hardly affected by the choice of land supply elasticities (see Supplementary Table S6-S8).

**Figure 5:**
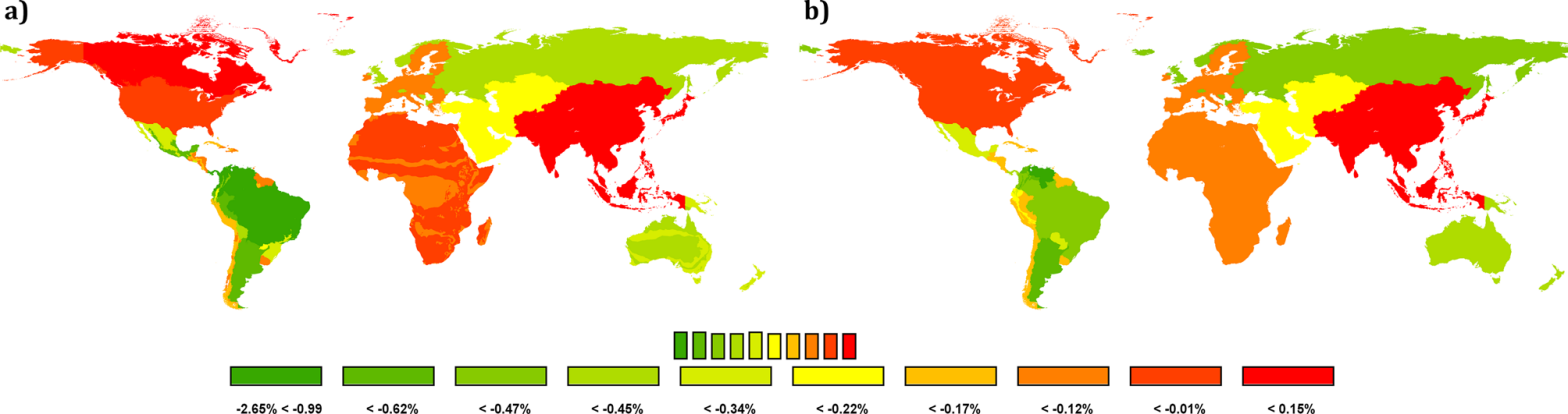
Percentage change in total managed land. Total managed land includes cropland, pasture, and managed forest. The changes are due to a dietary change (see “Materials and methods”). Map (**a**) depicts the calculated changes using the estimated elasticities from our econometric specification (Model (3)) and 0.05 for observations without estimates. Map (**b**) uses a standard elasticity of 0.05 for all observations.

As expected, the experiment reduces pasture, by around 3.3% or around 110 Mill. ha globally. Reflecting lower returns to pasture and higher demand for fruits and vegetables, crop lands increase globally by around 6% or 90 Mill. ha, while the total stock of managed land shrinks by 17 Mill. ha or −0.27%. These results based on the estimated elasticities change considerably if the default assumption of a land elasticity of 0.05% is used instead: the global reduction in managed land reduces from 17 to 12 Mill. ha despite similar changes in crop and pasture. These differences are depicted spatially in Fig. 5, which illustrates the percentage changes in managed land across mesoregions using either strategy. The difference in managed land stems mainly from lower expansion of managed forest when the estimated elasticities are used, with an expansion of 2.6 Mill. ha compared to 4.4 Mill. ha under a default elasticity assumption of 0.05%. Accordingly, with the estimated elasticities, the increased in unmanaged land is higher, for instance, unmanaged forest increased by 9.1 Mill. ha compared to 5 Mill. ha with the uniform 0.05% elasticity (see Supplementary Table S9).

## Discussion

In this study, we investigated key economic mechanisms behind agricultural expansion, the most important driver of deforestation in the tropics. Our main contribution is to quantify the sensitivity of agricultural expansion (and therefore forest loss) to commodity prices and governance quality in countries and agro-ecological zones across the global tropics. In doing so we address a major knowledge gap in the way of effectively safeguarding tropical forests as major providers of globally and locally valued ecosystem services. Three sets of results stand out. First, agricultural commodity prices are, on average, positively associated with land expansion, but this effect is quite heterogenous across the tropics. Consistent with theoretical expectations, we found that forest abundant regions are more prone to reallocate (forest) land to agricultural production when prices soar. This result is particularly strong in areas exposed to agribusiness and international markets, such as Brazil and Indonesia. In areas with high prevalence of shifting and subsistence agriculture, the distribution in the sensitivity of land supply to changes in agricultural prices varies such as Central Africa. In densely populated areas with large shares of agricultural land, land supply tends to be inelastic, which implies low flexibility of these agricultural systems to expand agricultural land.

Comparing our subnational elasticities with previous analyses, we found similar or smaller magnitudes of land supply elasticities (See Supplementary Table S10). For instance, Barr et al.^27^ calculated land supply elasticities for Brazil and found that they range from 0.66 to 0.89. When they included pasture, they obtained values ranging from 0.20 to 0.24 for Brazil, similar to our values ranging from 0.174 to 0.24. Villoria and Liu^25^ found lower magnitudes of land supply elasticities for their analysis of the Americas than the ones we calculated in our study. Liu and Villoria^29^ calculated elasticities for Sub-Saharan Africa and found highly inelastic values, which correspond to our relative small values across Africa. Compared with the land supply elasticities reported in Appendix B in the study by Tabeau et al.^26^, we found some strong similarities for some elasticities reported at the national level, such as those in Mexico (0.103), but there were high discrepancies with some of them, such as those in Indonesia (0.602) or Viet Nam (0.917). Some of the deviations in the results of various analyses are due to differences in the research period, indicators used, and scope of analysis. These studies used data from the 1990s and early 2000s to draw their estimations, while we used information from 2004 to 2015 in our calculations. Furthermore, it is noteworthy that during our timeframe of analysis, in Brazil, there was a significant change in the conservation policy regime, which led to lower expansion rates of deforestation^42–44^. Moreover, we used agricultural land including pastures instead of only cropland as the outcome variable. And lastly, we used a commodity price index while many other studies approximated the effect of land rents through cost-to-market accessibility or derived land rentability. A major strength of our analysis is that it uses a unified framework applied to a panel data structure to estimate elasticities in global tropical zones.

The second set of results pertains to the role of governance in changes in land supply. Our econometric estimations reveal a systematic negative relationship between stronger environmental governance and agricultural land expansion. We also observed that conventional indicators of governance are positively related to land supply. RoL seems to have a stronger effect on land supply increments among these conventional indicators. However, the systematic effect of conventional governance on land supply is not conclusive based on the statistical significance of the parameters calculated. These results are similar to those found by Ceddia et al.^50^ for tropical South America. They also used agricultural land as a measurement of land supply in their analysis. Our results indicate that not all the effects are endemic to South America but go beyond other areas of the tropics, especially the effect of environmental governance, which is a proxy for terrestrial biome protection.

The third set of results takes, out of the shelf, a global CGE model to evaluate how our land supply elasticities can affect assessments with this type of economic models. We simulate a shift from animal-based food towards a more vegetarian diet globally. Results using our elasticities did not alter the results in terms of supply and demand indicators compared to using uniform land supply elasticities of 0.05 as in previous applications. However, significant differences emerge in terms of land use change, i.e. a more pronounced reduction of managed land when utilizing our estimated elasticities. Our empirically estimated land supply elasticities thus potentially contribute to making the behavior of economic impact assessment models more realistic.

We note the following caveats of our analysis, which can be addressed in future research and as more data becomes available. First, we calculated land supply elasticities of commodity prices instead of land rents. Commodity prices are just one element of land rents and may not reflect regionally heterogeneous input scarcities. Better and more consistent subnational data is needed to capture variation in variable agricultural input costs.

Second, we rely on governance indicators at the national level, which implies averaging over important subnational variation in the quality of governance, especially in large countries with decentralized governance structures. Abstracting from the small magnitude of our effect estimate for environmental governance, our results confirm that it has a role to play in steering the global food and biomass systems towards sustainable outcomes. This interpretation is in line with an increasing number of subnational counterfactual-based studies quantifying the effectiveness of environmental policies on agricultural deforestation^51^. The lack of robustness here is likely due to the lack of globally consistent data on sub-national variation in governance quality. National level governance indicators cannot capture relevant heterogeneities in vertical governance structures, for example, related to (a) decision-making processes, (b) rules and policies, and (c) enforcement^38^. In fact, national-level governance indicators are likely biased against the agricultural frontier regions, where variation in land cover change is highest^52^. Third, from an application viewpoint, the incomplete region coverage is regrettable. Future research could focus on moderate zone regions, even if only to show that zero or close to zero land supply elasticities are a reasonable choice.

These caveats notwithstanding, our analysis has produced consistent estimates of land supply elasticities across the global tropics. Our results can inform the design of globally coherent land and forest conservation policies and enhance the ability of global economic models to assess related tradeoffs in alternative future policy scenarios.

## Materials and methods

### Data processing

**Figure 6:**
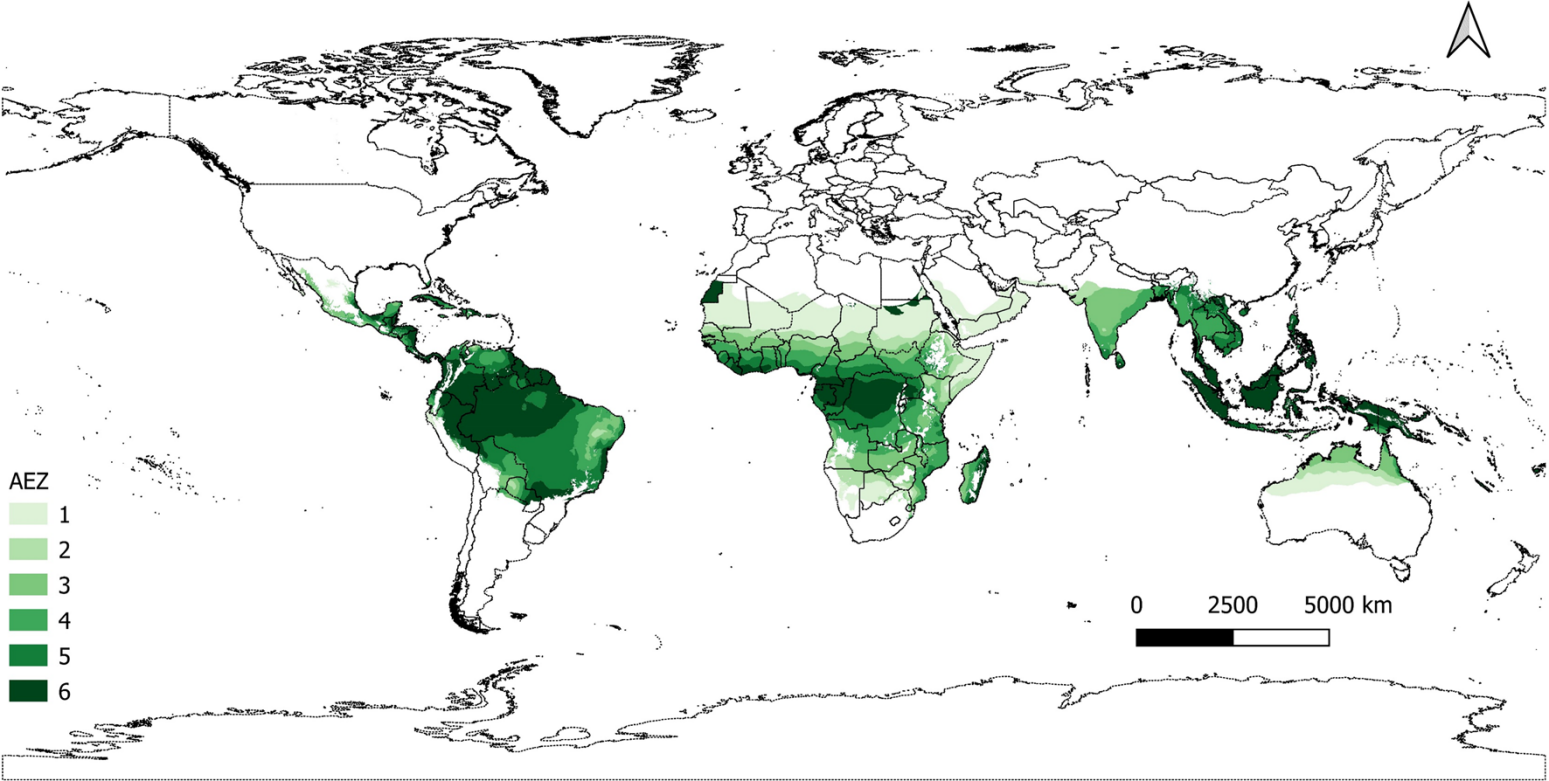
Tropical AEZ-country regions. Each mesoregion refers to the intersection between agro-ecological zones and national administrative boundaries. Shades of green reflect the different tropical zones considered for data analysis. We do not include those with a desert climate in the final econometric estimation, i.e., AEZ 1.

We conducted a subnational analysis using a mesoregion as the unit of observation. We refer to these mesoregions as agro-ecological zone (AEZ)-country units. These regions are obtained by intersecting spatial information on the Global AEZs (GAEZ; https://gaez.fao.org/)^53^ and national administrative boundaries for the whole world from the Database of Global Administrative Areas (https://gadm.org/index.html). Agro-ecological zones are regions with similar natural features like climate, soil, and terrain, influencing crop-specific agricultural potential. We identified 876 AEZ-country regions (see Fig. S1 for a graphic representation) based on the overview of GAEZ by Plevin et al.^54^, from which we selected tropical areas. We chose these AEZ-country observations as they have been subject to deforestation in the current century (visit https://globalforestwatch.org/ for a spatially explicit assessment on deforestation). Finally, 267 mesoregions were identified, as depicted in Fig. 6. The fundamental reason for using this level of analysis is that mesoregions are the units used in several applications of global CGE models such as those in the GTAP family^22,55,56^. Thus, the calculation of individual elasticities for these units of observation offers information to calibrate land use change in these CGE models (Fig. 6).

We measured *agricultural land*, which is the outcome variable in the empirical analysis, as the share of the total land in an AEZ-country observation. It is common to use only cropland as a response variable to analyze drivers of land supply and calculate its elasticities^25,27,50^. As cattle production is expanding in many in tropical zones, we include pastures in our measure of agricultural land expansion^32,57,58^. We used publicly available land cover information at ~ 300 m pixel resolution from the European Space Agency’s Climate Change Initiative for 2004–2015. These years are also covered in the GTAP v10A database for 2004, 2007, 2011, and 2014 (Aguiar et al.^59^). The land use cover database is publicly available at http://www.esa-landcover-cci.org/. This land cover data provides 22 land cover categories consistent at the global level (see Supplementary Table S1), from which we identify all pixels with categories related to cropland, pasture, forest, and other uses (residual category). We matched these pixels with their corresponding AEZ-country region so that we can add them up to obtain the number of km^2^ of agricultural land per mesoregion. Next, we used geometry tools from a geographic information system (GIS) to calculate the total area of each region. Finally, we calculated the ratio of agricultural land to the total area, resulting in our measure of agricultural land share per region.

We used a set of covariates that affect the socioeconomic, biophysical, and governance components of land rents. Among the set of socioeconomic variables, we mainly focus on the *agricultural commodity price index*. The index considers cereal crops, fibre crops, oil crops, pulses, roots and tubers, stimulants, sugar crops, fruit crops, vegetables, and animal-based commodities. It is important to note that our commodity price indicator includes agricultural commodities (except wood fiber) that are related to deforestation in tropical regions^46^. All commodities considered are presented in the Supplementary Table S2. To construct the index, we used national-level information on farm-gate prices provided by FAOSTAT(https://www.fao.org/faostat/en/#home). We used a methodology similar to FAO’s Laspeyres index with the average value of 2004 to 2006 as the base year to construct individual crop prices, which then were combined in our nine commodity groups using value-weights. Further, we used land suitability shares for these groups to downscale and combine them in our price index at the mesoregional level. Land suitability maps were obtained from FAO’s FGGD (https://data.apps.fao.org/catalog/organization/about/fao-food-insecurity-poverty-and-environment-global-gis-database-fggd) and produced by van Velthuizen et al.^60^. Land suitability is determined by comparing potential yields to maximum biological yield under optimal conditions, considering intermediate inputs and rainfed land availability. We use these land suitability shares to create a shift-share (Bartik) instrument as proposed by Goldsmith-Pinkham et al.^61^ and applied empirically to impacts on deforestation by Berman et al.^19^. This approach relies in the exogeneity of land suitability shares to agricultural land expansion. As mentioned by Berman et al.^58^, forest, and in our case agricultural land, may correlate with absolute land suitability, yet agricultural land expansion is likely exogenous to relative suitability for specific commodities. With this approach, we expect less impacts from possible price endogeneities with land use change outcomes. Finally, we deflated the index using an agriculture value-added deflator proposed by FAOSTAT. In the empirical model, we used the average value of the previous three years of each cross section to represent rationale price expectations^62^ and to further reduce endogeneity of prices. A more detailed description of our calculations is presented in the supplementary material.

We included additional indicators that affect the socioeconomic component of land rents. *Population density* (Pop) in each mesoregion is included to capture its potential role in the profitability and use of land resources. We employed the spatial estimation of the total population available on the WorldPop project (https://www.worldpop.org). This is a global rasterized layer at 100 m resolution in which each pixel offers a measure of the population count. We summed up the pixel information at the mesoregional level and divided it by the total area. In the empirical application, we included its quadratic transformation. We also tested different indicators of *fertilizer* use, namely the average national use of nitrogen, phosphorus (*P2O5*), and potassium per hectare obtained from FAOSTAT. Due to the high correlation among these indicators, we reported the results from the P205 indicator. The ratio of the agricultural sector ‘s value-added *exports and imports* (X/M) is included to control for trade effects on agricultural land use. The data on this variable are obtained also from FAOSTAT, measured at the national level, and like our commodity price index variable, we used the previous three years ‘ average in the analysis for each cross-sectional point. Unlike the commodity price, we could not downscale the fertilizer use and the trade indicators due to a lack of disaggregated sectoral information, so they are included at the national level.

We considered two indicators that affect the biophysical component of land rentability—g*rowing season length* (GSL) and *rain above 20 mm* (R20 mm) measured in days per year. These are climate extreme indices disaggregated at $0.25^\circ \times 0.25^\circ$ pixel resolution obtained from Mistry^63^ (https://doi.pangaea.de/10.1594/PANGAEA.898014). We aggregated these indicators at the mesoregion level by calculating the yearly average of pixels in a unit of observation.

Conventional and environmental governance indicators are also part of the covariates that account for the governance component. As conventional indicators, we tested the World Bank’s national perception indicators on corruption, RoL, and V&Acc (https://databank.worldbank.org/source/worldwide-governance-indicators). *V&Acc* denotes the degree to which citizens can participate in their government’s decisions, freedom of expression, freedom of association, and free media*; control of corruption* reflects the extent to which public officials use their power for private gain and when a state is overcome by elites and private interests; *RoL* indicates the ability to enforce contracts and the security of property rights^64^. As a proxy for environmental governance, we employ a *terrestrial biome protection index* (TBN), which is part of the environmental performance index prepared by the YALE Center for Environmental Law and Policy (https://epi.yale.edu/). The TBN reflects the size of protected area per biome type within national boundaries weighted by the prevalence of each biome in a country. This indicator evaluates a country's achievement in reaching Aichi Target 11 (established by the UN Convention of Biological Diversity in 2011), i.e., 17% protection for all biomes within its borders^65^.

### Fractional response model applied to land supply

We aim to explore how indicators linked to land rentability impact the primary cause of deforestation in tropical forests—specifically, the expected agricultural land allocation within a mesoregion (see “Data processing” above for details on our unit of analysis). Thus, we focus on calculating the sensitivity of agricultural land shares to changes in indicators that affect land rentability, i.e., land supply elasticities.

A linear function is one of the approaches that can be used to empirically estimate the effect of observed covariates on the outcome of interest. The use of standard linear models to test the effect of covariates on a fractional response variable, as in our present analysis, is seen as inappropriate in empirical approaches^48,66–68^. First, a linear functional form on the conditional mean of the response variable does not account for important nonlinearities inherent in a truncated and continuous variable^48,69^. Further, it is a common practice to use linear functional forms on logarithmic transformations of the response and covariates to determine elasticities. However, this poses difficulties for corner values of the outcome variable^48^. Third, linear models describe a misleading behavior of the outcome variable as there is no restriction on the range of values obtained from the structural function that relates the outcome with the observable covariates and unobservable heterogeneity^70^. In addition, a linear model needs a specification that can disentangle the individual effects from the global (average) effect on the sample to determine individual elasticities. One possibility is to include an interaction between the relevant covariate (i.e., our focus indicator of land rentability) and the individual fixed effects in the model. However, in our analysis, this would increase the likelihood of overfitting the model as the ratio of the number of observations to the number of predictors becomes smaller^71^. For these reasons, we decide to use the nonlinear model for a fractional outcome proposed by Papke and Wooldridge^48^ (P&W hereafter). P&W’s model proposed that an outcome variable is continuous but bounded to the range of 0 to 1. It is suitable for panel data structures and explicitly models time-constant unobserved heterogeneity. Furthermore, it is easy to empirically implement in conventional statistical programs. More complex models such as an exponential fractional model could have been used to relate our covariates to agricultural shares, but they require complex transformations of the estimations to determine the elasticities for each individual observation in the data (see^70^ for an example of these type of models). Moreover, they are hard to compare with other specifications.

### Econometric specification

P&W’s model starts with modeling the expectation of a fractional response variable ${y}_{it}$ conditional on a set of *K* explanatory observed variables ${\mathbf{x}}_{it}$ and an unobserved individual effect ${c}_{i}$ for *N* individuals in *T* time steps, in which $i=1, \dots , N$ and $t=1, \dots , T$. Following P&W, the conditional expectation takes the following form:

1 $$E\left({y}_{it}|{\mathbf{x}}_{it},{c}_{i}\right)=\Phi \left({\mathbf{x}}_{it}{\varvec{\upbeta}}+{c}_{i}\right), {\text{for}} i=1,\dots ,N;t=1,\dots , T,$$

where $\Phi \left(\bullet \right)$ represents the standard normal cumulative distribution function, and ${\varvec{\upbeta}}$ is a vector of *K* coefficients to be estimated. In this study, the set of covariates includes variables related to socioeconomic, biophysical, and governance (see details in “Data processing” above). The term ${c}_{i}$ represents the time-invariant individual unobserved heterogeneity.

Some useful properties of the normal function that help to derive partial effects and elasticities are that it is strictly monotonic, continuously differentiable, and nonadditively separable^70^. In particular, the monotonic property allows the elements of ${\varvec{\upbeta}}$ to give the direction of the partial effects of each covariate on the outcome variable^48^.

P&W included two additional assumptions to identify ${\varvec{\upbeta}}$ and the partial effects of relevant covariates. First, conditional on the unobserved heterogeneity, ${\mathbf{x}}_{it}$ in $t=1,\dots , T$ is strictly exogenous. This implies that $E\left({y}_{it}|{\mathbf{x}}_{i},{c}_{i}\right)=E\left({y}_{it}|{\mathbf{x}}_{it},{c}_{i}\right)$ for $t=1,\dots , T$, where ${\mathbf{x}}_{i}$ comprises the set of covariates in all periods. Second, the distribution of the unobserved heterogeneity ${c}_{i}$ is assumed to behave as a normal distribution conditional on the set of covariates such that

2 $${c}_{i}|({\mathbf{x}}_{i1},{\mathbf{x}}_{i1},\dots ,{\mathbf{x}}_{iT})={\text{Normal}}\left(\alpha +{\overline{\mathbf{x}} }_{i}{\varvec{\updelta}},{\sigma }_{u}^{2}\right)$$

where ${\overline{\mathbf{x}} }_{i}\equiv {T}^{-1}\sum_{t=1}^{T}{\mathbf{x}}_{it}$ represents the time averages of the time-varying covariates in the model. We also assume that ${u}_{i}\equiv {c}_{i}-\alpha -{\overline{\mathbf{x}} }_{i}{\varvec{\updelta}}$. These assumptions imply that ${u}_{i}$ conditional on $({\mathbf{x}}_{i1},{\mathbf{x}}_{i1},\dots ,{\mathbf{x}}_{iT})$ is also a normal distribution with a mean of 0 and a conditional variance, where ${\sigma }_{u}^{2}={\text{Var}}\left({c}_{i}|{\mathbf{x}}_{i}\right)$. P&W’s model is known as a correlated random effects approach as it allows correlation between unobserved effects and covariates using the Chamberlain–Mundlak strategy^69,72,73^.

Combining the previous assumptions and integrating them into Eq. (1), P&W demonstrated that both scaled elements of ${\varvec{\upbeta}}$ and partial effects are identified as long as the covariates have some time variability, and perfect linear relationships do not exist among them. This yields a model such that

3 $$E\left({y}_{it}|{\mathbf{x}}_{it},{u}_{i}\right)=\Phi \left(\alpha +{\mathbf{x}}_{it}{\varvec{\upbeta}}+{\overline{\mathbf{x}} }_{i}{\varvec{\updelta}}+{u}_{i}\right)$$

4 $$E\left({y}_{it}|{\mathbf{x}}_{i}\right)=E\left[\Phi \left(\alpha +{\mathbf{x}}_{it}{\varvec{\upbeta}}+{\overline{\mathbf{x}} }_{i}{\varvec{\updelta}}+{u}_{i}\right)|{\mathbf{x}}_{i}\right]=\Phi \left(\left[\alpha +{\mathbf{x}}_{it}{\varvec{\upbeta}}+{\overline{\mathbf{x}} }_{i}{\varvec{\updelta}}\right]/{[1+{\sigma }_{u}^{2}]}^{1/2}\right)$$

5 $$E\left({y}_{it}|{\mathbf{x}}_{i}\right)\equiv\Phi \left({\alpha }_{u}+{\mathbf{x}}_{it}{{\varvec{\upbeta}}}_{u}+{\overline{\mathbf{x}} }_{i}{{\varvec{\updelta}}}_{u}\right)$$

In Eq. (5), the parameters to be estimated has a subscript *u*, which refers to their transformation with a common scaling factor (${[1+{\sigma }_{u}^{2}]}^{1/2}$).

One major advantage of the P&W approach is that it avoids the need to use fixed effects on the empirical specification, which can cause an incidental parameters problem when *N* is large and *T* is small, as in our case^74,75^. Moreover, when the assumptions described above hold, this approach offers an option to calculate individual elasticities without explicitly modeling individual fixed effects.

### Individual elasticities

From our set of *K* covariates, we are primarily interested in the effect of agricultural commodity prices on land supply. The aim is to calculate individual elasticities of land supply (i.e., per observation) to agricultural prices. The first step is to determine the marginal effects from the empirical econometric model for each unit of observation. We used an approach similar to the average partial effects proposed for discrete response models^47^. However, instead of averaging at the cross section, we found the average for each $i$ in $T$ years. We multiplied the marginal effect with the ratio of the average value of the covariate of interest, that is, agricultural commodity price, and the average fitted value of the share of agricultural land per individual observation, ${\overline{x} }_{ki}/{\overline{\widehat{y}} }_{i}$. The calculation of each data point takes the following form:

6 $$\frac{\partial E\left({y}_{it}|{\mathbf{x}}_{i}\right)}{\partial {x}_{kit}}={\beta }_{ku}\times \phi \left({\alpha }_{u}+{\mathbf{x}}_{it}{{\varvec{\upbeta}}}_{u}+{\overline{\mathbf{x}} }_{i}{{\varvec{\updelta}}}_{u}\right)$$

from which a time-averaged individual land supply elasticity is computed as follows:

7 $${\varepsilon }_{i}=\frac{\partial E\left({y}_{i}|{\mathbf{x}}_{i}\right)}{\partial {x}_{ki}}\times \frac{{\overline{x} }_{ki}}{{\overline{\widehat{y}} }_{i}}={\beta }_{ku}\times \frac{{\sum }_{t=1}^{T}\phi \left({\alpha }_{u}+{\mathbf{x}}_{it}{{\varvec{\upbeta}}}_{u}+{\overline{\mathbf{x}} }_{i}{{\varvec{\updelta}}}_{u}\right)}{T}\times \frac{{\overline{x} }_{ki}}{{\overline{\widehat{y}} }_{i}}$$

where ${\beta }_{ku}$ is the estimated scaled coefficient related to variable $k$, e.g., commodity price; $\phi \left(\bullet \right)$ is the probability density function; and $\widehat{y}$ is the fitted value of the response variable. The symbol “¯” reflects average values for the research period.

### Comparative-static CGE analysis

To demonstrate the practical application of the estimated elasticities, we undertake a comparative-static analysis using Computable General Equilibrium (CGE) techniques. This analysis investigates the effects of a preference shift globally from animal-based foods to a more vegetarian diet. Specifically, we adjust demand system parameters so that, at constant prices and incomes, households decrease their calorie consumption from a combination of meat, fish, and dairy products by 25%. In contrast, they increase their calorie intake from fruits, vegetables, vegetable oils, and 25% of other food products.

We construct a data base with full commodity and activity detail from the GTAP V11 Data Base^76^, keeping regions in North and South America as separate countries (USA; Mexico, Argentina, Brazil, Bolivia, Venezuela, San Salvador, Paraguay, Ecuador, Jamaica, Dominican Republic) and aggregating others into regional aggregates (Africa, EU, Oceania, East Asia, Rest of Europe, Rest of World). The simulations are conducted in CGEBox^20^, using an MAIDADS demand system as estimated by Britz^77^. The model set-up includes an extended version of the GTAP-AEZ model^78^ where the stock of total land in economic use is not fixed, but reacts to changes in land rents. The shock is compared under two set of parameters (1) the estimated elasticities for country-AEZ combinations, where available, and default supply elasticities of land 0.05 elsewhere, (2) a land supply elasticity of 0.05 for all model regions and AEZs.

The model uses nests in the production functions and in final demand as described in Wilts and Britz^79^ and follows otherwise the layout of the GAMS model version 7^80^.

### Supplementary Information

Supplementary Information.

## Data Availability

All data is from public sources, as detailed in the manuscript.
