# Supplementary material for: Impacts of commodity prices and governance on the expansion of tropical agricultural frontiers
Source: Sci Rep. 2024 Apr 22;14:9209. doi: 10.1038/s41598-024-59446-0 (PMC11035705; doi:10.1038/s41598-024-59446-0)
Supplement: Supplementary file 1 — Supplementary Information. [file 41598_2024_59446_MOESM1_ESM.docx]

Supplementary Information for

Impacts of commodity prices and governance on the expansion of tropical agricultural frontiers

Javier Miranda ^1*^, Wolfgang Britz ^1^, Jan Börner ^1,2^

^1^ *Institute for Food and Resource Economics, University of Bonn, Nussallee 21, 53115 Bonn, Germany*

^2^ *Center for Development Research, University of Bonn, Genscherallee 3, 53113 Bonn, Germany*

^*^Corresponding author: Institute for Food and Resource Economics, University of Bonn, Nussallee 21, 53115 Bonn, Germany

*E-mail address*: [javier.miranda@ilr.uni-bonn.de](mailto:javier.miranda@ilr.uni-bonn.de)

Commodity price index at the subnational level

We used the FAOSTAT database to obtain information on individual commodity prices to create our index. We then utilized the available land cover information within each AEZ-country region to obtain an index at the subnational level. The agricultural commodities included are related to three aggregated groups identified by FAO—primary crops, meat, and milk products (see Supplementary Table S2).

Our starting point is to obtain subindices that account for the importance of each commodity in the agricultural sector. For this, we calculated weights, $\omega_{iat}$, representing each commodity’s (*i*) importance in the total value of agricultural production ($V_{at}$). This calculation is done separately for each aggregated group of products (*a*), i.e., cereals, fibre, oil, pulses, roots and tubers, stimulants, sugar, fruit, vegetables, and animal-based commodities, at each time step *t*. All the weights for each group of commodities, $\omega_{iat}$, sum to unity. For each commodity, we multiplied the weights with the ratio of current prices to the price of our base year (i.e., average of 2004 to 2006). We then summed all prices to obtain a single price for each commodity group *a*. These steps are represented in Equations S1.1 to S1.4.

$V_{at}=\sum_{i=1}^{N} V_{iat}$ (S1.1)

$\omega_{iat}=\frac{V_{iat}}{V_{at}}, and \sum_{i=1}^{N} \omega_{iat}=1$ (S1.2)

${wP}_{iat}=\frac{P_{iat}}{P_{iab}}*\omega_{iat}$ (S1.3)

${wP}_{at}=\sum_{i=1}^{N} {wP}_{iat}$ (S1.4)

Our next set of steps involves the calculation of the price index at the subnational level. For this, we used land suitability share weights that reflect the potential for each group of crops *a* in each mesoregion. We used gridded maps obtain from FAO’s FGGD (<https://data.apps.fao.org/catalog/organization/about/fao-food-insecurity-poverty-and-environment-global-gis-database-fggd>). We need to aggregate the information at our level of analysis. To achieve this, we calculate the median land suitability values for each region, ${LS}_{a}$. We then calculate each share ($\alpha_{a}$) with the ratio of the respective land suitability to the sum of all suitability values. We multiplied each share to its corresponding price. Finally, we sum up the values obtained and multiplied by 100 to obtain our index for agricultural commodities. We summarize these steps in Equations S1.5 and S1.6 as follows:

$\alpha_{a}=\frac{{LS}_{a}}{\sum_{a=1}^{N} {LS}_{a}}, and \sum_{a=1}^{N} \alpha_{a}=1$ (S1.5)

$P_{t}=(\sum_{a=1}^{N} \alpha_{a}*wP_{at})*100$ (S1.6)

Finally, we convert the subnational agricultural price index to real terms by dividing it with the agricultural deflator in the FAOSTAT database.

$RealP_{t}=\frac{P_{t}}{{AgrDefl}_{t}}$ (S1.7)

Our final price index, $RealP_{t}$, is then used to calculate a three-year average for each year (*t*), from *t*-1 to *t*-3. This is the final variable used in the econometric analysis, and it was used to calculate the land supply elasticities reported in the main text.

Table S1. Aggregated Land Cover classes

| **Aggregated Classes** | **Code in Raster** | **Pixel Categories** |
| --- | --- | --- |
| No Data | 0 | No Data |
| Agriculture | 10 | Rainfed cropland |
|  | 11 | Rainfed cropland |
|  | 12 | Rainfed cropland |
|  | 20 | Irrigated cropland |
|  | 30 | Mosaic cropland/vegetation |
|  | 40 | Mosaic cropland/vegetation |
|  | 110 | Mosaic herbaceous/tree and shrub |
|  | 130 | Grassland |
| Forest | 50 | Tree cover, broadleaved, evergreen |
|  | 60 | Tree cover, broadleaved, deciduous |
|  | 61 | Tree cover, broadleaved, deciduous |
|  | 62 | Tree cover, broadleaved, deciduous |
|  | 70 | Tree cover, needleleaved, evergreen |
|  | 71 | Tree cover, needleleaved, evergreen |
|  | 72 | Tree cover, needleleaved, evergreen |
|  | 80 | Tree cover, needleleaved, deciduous |
|  | 81 | Tree cover, needleleaved, deciduous |
|  | 82 | Tree cover, needleleaved, deciduous |
|  | 90 | Tree cover, mixed leaf type |
|  | 100 | Mosaic tree and shrub / herbaceous cover |
|  | 160 | Tree cover, flooded, fresh or brakish water |
|  | 170 | Tree cover, flooded, saline water |
| Other Land Uses | 140 | Lichens and mosses |
|  | 150 | Sparse vegetation |
|  | 151 | Sparse vegetation |
|  | 152 | Sparse vegetation |
|  | 153 | Sparse vegetation |
|  | 200 | Bare areas |
|  | 120 | Shrubland |
|  | 121 | Shrubland |
|  | 122 | Shrubland |
|  | 220 | Snow |
|  | 190 | Urban |
|  | 210 | Water |
|  | 180 | Shrub or herbaceous cover, flooded, fresh-saline or brakish water |

These are the codes from the ESA-CCI-LC from which we obtained the dependent variable. The sum of all pixels in the land cover data within an AEZ country classified as agriculture in the table constitute the total land devoted to agriculture. We divided this information with the total area of the region (calculated using GIS tools) to obtain our agricultural land use share per unit of each year observation.

Table S2. Agricultural commodities considered

| **FAO ID** | **Commodity** | **Commodity Group** |  | **FAO ID** | **Commodity** | **Commodity Group** |
| --- | --- | --- | --- | --- | --- | --- |
| 44 | Barley | Cereal crops |  | 277 | Jojoba seed | Oil crops |
| 89 | Buckwheat | Cereal crops |  | 263 | Karite nuts (sheanuts) | Oil crops |
| 101 | Canary seed | Cereal crops |  | 333 | Linseed | Oil crops |
| 94 | Fonio | Cereal crops |  | 299 | Melonseed | Oil crops |
| 56 | Maize | Cereal crops |  | 292 | Mustard seed | Oil crops |
| 79 | Millet | Cereal crops |  | 254 | Oil palm fruit | Oil crops |
| 75 | Oats | Cereal crops |  | 339 | Oilseeds nes | Oil crops |
| 92 | Quinoa | Cereal crops |  | 260 | Olives | Oil crops |
| 27 | Rice, paddy | Cereal crops |  | 296 | Poppy seed | Oil crops |
| 71 | Rye | Cereal crops |  | 270 | Rapeseed | Oil crops |
| 83 | Sorghum | Cereal crops |  | 280 | Safflower seed | Oil crops |
| 97 | Triticale | Cereal crops |  | 328 | Seed cotton | Oil crops |
| 15 | Wheat | Cereal crops |  | 289 | Sesame seed | Oil crops |
| 800 | Agave fibres nes | Fibre crops |  | 236 | Soybeans | Oil crops |
| 782 | Bastfibres, other | Fibre crops |  | 267 | Sunflower seed | Oil crops |
| 821 | Fibre crops nes | Fibre crops |  | 305 | Tallowtree seed | Oil crops |
| 773 | Flax fibre and tow | Fibre crops |  | 275 | Tung nuts | Oil crops |
| 777 | Hemp tow waste | Fibre crops |  | 203 | Bambara beans | Pulses |
| 780 | Jute | Fibre crops |  | 176 | Beans, dry | Pulses |
| 310 | Kapok fruit | Fibre crops |  | 181 | Broad beans, horse beans, dry | Pulses |
| 809 | Manila fibre (abaca) | Fibre crops |  | 191 | Chick peas | Pulses |
| 788 | Ramie | Fibre crops |  | 195 | Cow peas, dry | Pulses |
| 789 | Sisal | Fibre crops |  | 201 | Lentils | Pulses |
| 265 | Castor oil seed | Oil crops |  | 210 | Lupins | Pulses |
| 249 | Coconuts | Oil crops |  | 187 | Peas, dry | Pulses |
| 242 | Groundnuts, with shell | Oil crops |  | 197 | Pigeon peas | Pulses |
| 336 | Hempseed | Oil crops |  | 205 | Vetches | Pulses |

Table S2. Agricultural commodities considered (continue)

| **FAO ID** | **Commodity** | **Commodity Group** |  | **FAO ID** | **Commodity** | **Commodity Group** |
| --- | --- | --- | --- | --- | --- | --- |
| 125 | Cassava | Roots and Tubers |  | 541 | Fruit, stone nes | Fruit crops |
| 116 | Potatoes | Roots and Tubers |  | 603 | Fruit, tropical fresh nes | Fruit crops |
| 122 | Sweet potatoes | Roots and Tubers |  | 507 | Grapefruit (inc. pomelos) | Fruit crops |
| 136 | Taro (cocoyam) | Roots and Tubers |  | 560 | Grapes | Fruit crops |
| 137 | Yams | Roots and Tubers |  | 592 | Kiwi fruit | Fruit crops |
| 135 | Yautia (cocoyam) | Roots and Tubers |  | 497 | Lemons and limes | Fruit crops |
| 661 | Cocoa, beans | Stimulants |  | 571 | Mangoes, mangosteens, guavas | Fruit crops |
| 656 | Coffee, green | Stimulants |  | 568 | Melons, other (inc.cantaloupes) | Fruit crops |
| 826 | Tobacco, unmanufactured | Stimulants |  | 490 | Oranges | Fruit crops |
| 157 | Sugar beet | Sugar crops |  | 600 | Papayas | Fruit crops |
| 156 | Sugar cane | Sugar crops |  | 534 | Peaches and nectarines | Fruit crops |
| 515 | Apples | Fruit crops |  | 521 | Pears | Fruit crops |
| 526 | Apricots | Fruit crops |  | 587 | Persimmons | Fruit crops |
| 572 | Avocados | Fruit crops |  | 574 | Pineapples | Fruit crops |
| 486 | Bananas | Fruit crops |  | 489 | Plantains and others | Fruit crops |
| 558 | Berries nes | Fruit crops |  | 536 | Plums and sloes | Fruit crops |
| 552 | Blueberries | Fruit crops |  | 523 | Quinces | Fruit crops |
| 461 | Carobs | Fruit crops |  | 547 | Raspberries | Fruit crops |
| 591 | Cashewapple | Fruit crops |  | 544 | Strawberries | Fruit crops |
| 531 | Cherries | Fruit crops |  | 495 | Tangerines, mandarins, clementines, satsumas | Fruit crops |
| 530 | Cherries, sour | Fruit crops |  | 567 | Watermelons | Fruit crops |
| 554 | Cranberries | Fruit crops |  | 366 | Artichokes | Vegetables |
| 550 | Currants | Fruit crops |  | 367 | Asparagus | Vegetables |
| 577 | Dates | Fruit crops |  | 414 | Beans, green | Vegetables |
| 569 | Figs | Fruit crops |  | 358 | Cabbages and other brassicas | Vegetables |
| 512 | Fruit, citrus nes | Fruit crops |  | 426 | Carrots and turnips | Vegetables |
| 619 | Fruit, fresh nes | Fruit crops |  | 393 | Cauliflowers and broccoli | Vegetables |

Table S2. Agricultural commodities considered (continue)

| **FAO ID** | **Commodity** | **Commodity Group** |
| --- | --- | --- |
| 401 | Chillies and peppers, green | Vegetables |
| 397 | Cucumbers and gherkins | Vegetables |
| 399 | Eggplants (aubergines) | Vegetables |
| 406 | Garlic | Vegetables |
| 407 | Leeks, other alliaceous vegetables | Vegetables |
| 372 | Lettuce and chicory | Vegetables |
| 446 | Maize, green | Vegetables |
| 449 | Mushrooms and truffles | Vegetables |
| 430 | Okra | Vegetables |
| 403 | Onions, dry | Vegetables |
| 402 | Onions, shallots, green | Vegetables |
| 417 | Peas, green | Vegetables |
| 394 | Pumpkins, squash and gourds | Vegetables |
| 373 | Spinach | Vegetables |
| 423 | String beans | Vegetables |
| 388 | Tomatoes | Vegetables |
| 463 | Vegetables, fresh nes | Vegetables |
| 420 | Vegetables, leguminous nes | Vegetables |
| 947 | Meat, buffalo | Animal-based |
| 1127 | Meat, camel | Animal-based |
| 867 | Meat, cattle | Animal-based |
| 1017 | Meat, goat | Animal-based |
| 977 | Meat, sheep | Animal-based |
| 951 | Milk, whole fresh buffalo | Animal-based |
| 1130 | Milk, whole fresh camel | Animal-based |
| 882 | Milk, whole fresh cow | Animal-based |
| 1020 | Milk, whole fresh goat | Animal-based |
| 982 | Milk, whole fresh sheep | Animal-based |

Table S3. Data summary statistics

|  | | | | | |
| --- | --- | --- | --- | --- | --- |
|  | N | Mean | St. Dev. | Min | Max |
|  | | | | | |
| Agricultural land share | 2,112 | 0.375 | 0.251 | 0.0001 | 0.971 |
| Commodity price index^‡^ | 2,112 | 1.513 | 0.539 | 0.197 | 4.992 |
| Population density (#/km2) | 2,112 | 104.689 | 151.591 | 0.075 | 1,191.136 |
| Fertilizer use index | 2,112 | 18.092 | 21.484 | 0.000 | 108.850 |
| Growing Season Length (days/year) | 2,112 | 365.246 | 0.438 | 360.688 | 366.000 |
| Rain above 20 mm (days/year) | 2,112 | 138.824 | 56.536 | 0.675 | 304.619 |
| Ratio agricultural exports to Imports^‡^ | 2,112 | 1.084 | 0.799 | 0.124 | 10.306 |
| Rule of Law index | 2,112 | -0.217 | 0.648 | -1.749 | 1.507 |
| Corruption index | 2,112 | -0.490 | 0.591 | -1.497 | 2.052 |
| Voice and Accountability index | 2,112 | -0.517 | 0.618 | -2.032 | 1.923 |
| Terrestrial Biome Protection index | 2,112 | 70.461 | 26.692 | 0.664 | 100.000 |
| Year | 2,112 | 2,009.500 | 3.453 | 2,004 | 2,015 |
| ^‡^Statistics for the variable transformed as 3-years-rolling-average | | | | | |
|  | | | | | |

Table S4. Fractional response models

|  | | | |
| --- | --- | --- | --- |
|  | | | |
|  | *Dependent variable:* | | |
|  |  | | |
|  | Agricultural land share | | |
|  | (1) | (2) | (3) |
|  | | | |
| Commodity price | 0.076^a^ | 0.081^a^ | 0.080^a^ |
|  | (0.021) | (0.022) | (0.022) |
|  |  |  |  |
| Pop | 0.240^a^ | 0.216^a^ | 0.228^b^ |
|  | (0.082) | (0.080) | (0.089) |
|  |  |  |  |
| Pop^2^ | -0.013^a^ | -0.011^a^ | -0.012^a^ |
|  | (0.004) | (0.004) | (0.004) |
|  |  |  |  |
| Fertilizer use | 0.017 | -0.021 | 0.012 |
|  | (0.063) | (0.062) | (0.060) |
|  |  |  |  |
| GSL | 0.015^c^ | 0.014 | 0.016^c^ |
|  | (0.009) | (0.009) | (0.009) |
|  |  |  |  |
| R20 mm | -0.0003 | -0.0002 | -0.0002 |
|  | (0.0002) | (0.0002) | (0.0002) |
|  |  |  |  |
| X/M | -0.011 | -0.017 | -0.014 |
|  | (0.011) | (0.012) | (0.012) |
|  |  |  |  |
| V&Acc | 0.022 |  |  |
|  | (0.046) |  |  |
|  |  |  |  |
| Corr |  | 0.003 |  |
|  |  | (0.046) |  |
|  |  |  |  |
| RoL |  |  | 0.042 |
|  |  |  | (0.049) |
|  |  |  |  |
| TBN | -0.002^b^ | -0.002^a^ | -0.002^a^ |
|  | (0.001) | (0.001) | (0.001) |
| Time Effects | Yes | Yes | Yes |
| Regressor’s period mean^*^ | Yes | Yes | Yes |
| Observations | 2,112 | 2,112 | 2,112 |
|  | | | |

Columns 1–3 represent different estimations using V&Acc, Corruption, and RoL as conventional governance indicators, respectively. ^a^ Significant at the 1% level. ^b^ Significant at the 5% level. ^c^ Significant at the 10% level. ^∗^The P&W [1] model includes this set of covariates to control for unobserved heterogeneity. Robust standard errors clustered at the country level are presented in parentheses.

Table S5. Subnational elasticities

| **AEZ code** | **Country** | **(1) Mod. V&Acc** | **(2) Mod. Corr** | **(3) Mod. RoL** |
| --- | --- | --- | --- | --- |
| 3 | Argentina | 0,198 | 0,214 | 0,206 |
| 4 | Argentina | 0,199 | 0,216 | 0,208 |
| 5 | Argentina | 0,200 | 0,216 | 0,208 |
| 6 | Argentina | 0,201 | 0,217 | 0,210 |
| 2 | Australia | 0,122 | 0,158 | 0,136 |
| 3 | Australia | 0,131 | 0,168 | 0,146 |
| 4 | Australia | 0,141 | 0,180 | 0,157 |
| 5 | Australia | 0,140 | 0,180 | 0,156 |
| 4 | Bangladesh | 0,059 | 0,059 | 0,061 |
| 5 | Bangladesh | 0,090 | 0,093 | 0,094 |
| 5 | Belize | 0,113 | 0,113 | 0,110 |
| 6 | Belize | 0,120 | 0,120 | 0,118 |
| 3 | Bolivia | 0,126 | 0,134 | 0,129 |
| 4 | Bolivia | 0,143 | 0,152 | 0,148 |
| 5 | Bolivia | 0,157 | 0,167 | 0,162 |
| 6 | Bolivia | 0,166 | 0,177 | 0,172 |
| 2 | Brazil | 0,174 | 0,182 | 0,177 |
| 3 | Brazil | 0,176 | 0,185 | 0,180 |
| 4 | Brazil | 0,187 | 0,197 | 0,192 |
| 5 | Brazil | 0,207 | 0,217 | 0,213 |
| 6 | Brazil | 0,231 | 0,243 | 0,239 |
| 6 | Brunei | 0,117 | 0,150 | 0,141 |
| 2 | Burkina Faso | 0,086 | 0,098 | 0,092 |
| 3 | Burkina Faso | 0,083 | 0,095 | 0,089 |
| 4 | Burkina Faso | 0,101 | 0,114 | 0,108 |
| 4 | Burundi | 0,106 | 0,111 | 0,113 |
| 5 | Burundi | 0,091 | 0,096 | 0,098 |
| 4 | Cambodia | 0,119 | 0,124 | 0,127 |
| 5 | Cambodia | 0,109 | 0,114 | 0,117 |
| 2 | Cameroon | 0,067 | 0,070 | 0,071 |
| 3 | Cameroon | 0,063 | 0,066 | 0,067 |
| 4 | Cameroon | 0,097 | 0,101 | 0,104 |
| 5 | Cameroon | 0,104 | 0,109 | 0,111 |
| 6 | Cameroon | 0,099 | 0,104 | 0,106 |
| 4 | China | 0,019 | 0,023 | 0,020 |
| 5 | China | 0,107 | 0,118 | 0,120 |
| 6 | China | 0,094 | 0,105 | 0,106 |

Note: The table presents the calculated individual elasticities using the different specifications presented in Table SI.7. These specifications vary depending on the conventional governance indicator employed. The acronyms are defined as follows: V&Acc=voice and accountability; Corr=corruption; RoL=rule of law.

Table S5. Subnational elasticities (continue)

| **AEZ code** | **Country** | **(1) Mod. V&Acc** | **(2) Mod. Corr** | **(3) Mod. RoL** |
| --- | --- | --- | --- | --- |
| 2 | Colombia | 0,150 | 0,160 | 0,154 |
| 3 | Colombia | 0,107 | 0,115 | 0,109 |
| 4 | Colombia | 0,096 | 0,104 | 0,098 |
| 5 | Colombia | 0,169 | 0,180 | 0,174 |
| 6 | Colombia | 0,188 | 0,199 | 0,195 |
| 5 | Costa Rica | 0,161 | 0,181 | 0,167 |
| 6 | Costa Rica | 0,158 | 0,177 | 0,164 |
| 3 | Côte d'Ivoire | 0,100 | 0,110 | 0,109 |
| 4 | Côte d'Ivoire | 0,110 | 0,122 | 0,120 |
| 5 | Côte d'Ivoire | 0,110 | 0,122 | 0,120 |
| 6 | Côte d'Ivoire | 0,104 | 0,116 | 0,114 |
| 3 | Dominican Republic | 0,109 | 0,108 | 0,109 |
| 4 | Dominican Republic | 0,106 | 0,105 | 0,106 |
| 5 | Dominican Republic | 0,075 | 0,074 | 0,074 |
| 6 | Dominican Republic | 0,077 | 0,076 | 0,076 |
| 2 | Ecuador | 0,137 | 0,144 | 0,140 |
| 3 | Ecuador | 0,083 | 0,089 | 0,084 |
| 4 | Ecuador | 0,132 | 0,139 | 0,135 |
| 5 | Ecuador | 0,157 | 0,165 | 0,161 |
| 6 | Ecuador | 0,197 | 0,208 | 0,204 |
| 6 | Egypt | 0,112 | 0,122 | 0,127 |
| 4 | El Salvador | 0,069 | 0,073 | 0,067 |
| 5 | El Salvador | 0,110 | 0,115 | 0,109 |
| 2 | Ethiopia | 0,102 | 0,122 | 0,117 |
| 3 | Ethiopia | 0,101 | 0,121 | 0,116 |
| 4 | Ethiopia | 0,115 | 0,135 | 0,131 |
| 5 | Ethiopia | 0,106 | 0,126 | 0,121 |
| 5 | Fiji | 0,110 | 0,126 | 0,119 |
| 6 | Fiji | 0,106 | 0,122 | 0,114 |
| 3 | Gambia | 0,051 | 0,058 | 0,057 |
| 3 | Ghana | 0,083 | 0,091 | 0,086 |
| 4 | Ghana | 0,096 | 0,106 | 0,101 |
| 5 | Ghana | 0,100 | 0,110 | 0,105 |
| 6 | Ghana | 0,095 | 0,105 | 0,099 |
| 3 | Guinea | 0,091 | 0,098 | 0,096 |
| 4 | Guinea | 0,101 | 0,109 | 0,107 |
| 5 | Guinea | 0,116 | 0,125 | 0,123 |
| 5 | Guyana | 0,027 | 0,028 | 0,028 |
| 6 | Guyana | 0,0379 | 0,0382 | 0,0385 |

Table S5. Subnational elasticities (continue)

| **AEZ code** | **Country** | **(1) Mod. V&Acc** | **(2) Mod. Corr** | **(3) Mod. RoL** |
| --- | --- | --- | --- | --- |
| 4 | Honduras | 0,103 | 0,106 | 0,105 |
| 5 | Honduras | 0,104 | 0,107 | 0,106 |
| 6 | Honduras | 0,124 | 0,128 | 0,127 |
| 2 | India | 0,068 | 0,067 | 0,068 |
| 3 | India | 0,071 | 0,070 | 0,071 |
| 4 | India | 0,061 | 0,061 | 0,061 |
| 5 | India | 0,075 | 0,074 | 0,075 |
| 3 | Indonesia | 0,173 | 0,181 | 0,181 |
| 4 | Indonesia | 0,196 | 0,205 | 0,206 |
| 5 | Indonesia | 0,108 | 0,116 | 0,114 |
| 6 | Indonesia | 0,200 | 0,209 | 0,210 |
| 3 | Jamaica | 0,065 | 0,069 | 0,063 |
| 4 | Jamaica | 0,096 | 0,100 | 0,095 |
| 2 | Kenya | 0,126 | 0,125 | 0,128 |
| 3 | Kenya | 0,134 | 0,133 | 0,137 |
| 4 | Kenya | 0,118 | 0,117 | 0,121 |
| 5 | Kenya | 0,089 | 0,088 | 0,091 |
| 6 | Kenya | 0,101 | 0,099 | 0,102 |
| 2 | Madagascar | 0,149 | 0,169 | 0,160 |
| 3 | Madagascar | 0,171 | 0,193 | 0,184 |
| 4 | Madagascar | 0,183 | 0,206 | 0,197 |
| 5 | Madagascar | 0,185 | 0,208 | 0,199 |
| 6 | Madagascar | 0,191 | 0,216 | 0,206 |
| 3 | Malawi | 0,106 | 0,117 | 0,120 |
| 4 | Malawi | 0,117 | 0,128 | 0,132 |
| 6 | Malaysia | 0,187 | 0,227 | 0,219 |
| 2 | Mali | 0,084 | 0,085 | 0,085 |
| 3 | Mali | 0,086 | 0,088 | 0,088 |
| 4 | Mali | 0,088 | 0,090 | 0,091 |
| 2 | Mexico | 0,084 | 0,088 | 0,082 |
| 3 | Mexico | 0,100 | 0,105 | 0,101 |
| 4 | Mexico | 0,105 | 0,110 | 0,106 |
| 5 | Mexico | 0,111 | 0,116 | 0,112 |
| 6 | Mexico | 0,101 | 0,107 | 0,102 |
| 6 | Morocco | 0,076 | 0,086 | 0,084 |
| 2 | Mozambique | 0,097 | 0,103 | 0,101 |
| 3 | Mozambique | 0,088 | 0,093 | 0,091 |
| 4 | Mozambique | 0,091 | 0,096 | 0,095 |
| 5 | Mozambique | 0,091 | 0,097 | 0,095 |

Table S5. Subnational elasticities (continue)

| **AEZ code** | **Country** | **(1) Mod. V&Acc** | **(2) Mod. Corr** | **(3) Mod. RoL** |
| --- | --- | --- | --- | --- |
| 2 | Namibia | 0,096 | 0,112 | 0,103 |
| 3 | Namibia | 0,073 | 0,085 | 0,079 |
| 4 | Nicaragua | 0,095 | 0,101 | 0,099 |
| 5 | Nicaragua | 0,119 | 0,127 | 0,125 |
| 6 | Nicaragua | 0,135 | 0,143 | 0,142 |
| 2 | Niger | 0,075 | 0,079 | 0,078 |
| 3 | Niger | 0,081 | 0,085 | 0,084 |
| 2 | Nigeria | 0,056 | 0,057 | 0,057 |
| 3 | Nigeria | 0,060 | 0,061 | 0,062 |
| 4 | Nigeria | 0,072 | 0,073 | 0,075 |
| 5 | Nigeria | 0,051 | 0,053 | 0,053 |
| 6 | Nigeria | 0,056 | 0,059 | 0,059 |
| 5 | Panama | 0,135 | 0,141 | 0,139 |
| 6 | Panama | 0,130 | 0,135 | 0,133 |
| 2 | Paraguay | 0,168 | 0,164 | 0,173 |
| 3 | Paraguay | 0,151 | 0,147 | 0,155 |
| 4 | Paraguay | 0,152 | 0,148 | 0,156 |
| 5 | Paraguay | 0,165 | 0,161 | 0,170 |
| 6 | Paraguay | 0,163 | 0,159 | 0,167 |
| 2 | Peru | 0,129 | 0,140 | 0,133 |
| 3 | Peru | 0,134 | 0,145 | 0,137 |
| 4 | Peru | 0,174 | 0,187 | 0,180 |
| 5 | Peru | 0,174 | 0,188 | 0,181 |
| 6 | Peru | 0,164 | 0,177 | 0,168 |
| 4 | Philippines | 0,074 | 0,078 | 0,077 |
| 5 | Philippines | 0,072 | 0,077 | 0,075 |
| 6 | Philippines | 0,101 | 0,105 | 0,104 |
| 5 | Republic of Congo | 0,145 | 0,156 | 0,159 |
| 6 | Republic of Congo | 0,149 | 0,160 | 0,163 |
| 2 | Senegal | 0,061 | 0,067 | 0,064 |
| 3 | Senegal | 0,072 | 0,079 | 0,076 |
| 2 | South Africa | 0,112 | 0,124 | 0,116 |
| 3 | South Africa | 0,106 | 0,118 | 0,109 |
| 4 | South Africa | 0,105 | 0,116 | 0,108 |
| 5 | South Africa | 0,091 | 0,101 | 0,093 |
| 3 | Sri Lanka | 0,096 | 0,109 | 0,107 |
| 4 | Sri Lanka | 0,107 | 0,122 | 0,120 |
| 5 | Sri Lanka | 0,110 | 0,125 | 0,123 |
| 6 | Sri Lanka | 0,065 | 0,079 | 0,075 |

Table S5. Subnational elasticities (continue)

| **AEZ code** | **Country** | **(1) Mod. V&Acc** | **(2) Mod. Corr** | **(3) Mod. RoL** |
| --- | --- | --- | --- | --- |
| 6 | Suriname | 0,153 | 0,163 | 0,157 |
| 3 | Tanzania | 0,099 | 0,107 | 0,107 |
| 4 | Tanzania | 0,101 | 0,109 | 0,109 |
| 5 | Tanzania | 0,105 | 0,113 | 0,113 |
| 6 | Tanzania | 0,118 | 0,127 | 0,128 |
| 4 | Thailand | 0,116 | 0,134 | 0,131 |
| 5 | Thailand | 0,141 | 0,160 | 0,158 |
| 6 | Thailand | 0,141 | 0,160 | 0,158 |
| 3 | Togo | 0,075 | 0,083 | 0,083 |
| 4 | Togo | 0,079 | 0,086 | 0,087 |
| 5 | Togo | 0,086 | 0,094 | 0,095 |
| 6 | Trinidad and Tobago | 0,116 | 0,130 | 0,120 |
| 4 | United States | 0,123 | 0,151 | 0,135 |
| 5 | United States | 0,052 | 0,074 | 0,060 |
| 2 | Venezuela | 0,225 | 0,243 | 0,229 |
| 3 | Venezuela | 0,189 | 0,206 | 0,192 |
| 4 | Venezuela | 0,268 | 0,289 | 0,275 |
| 5 | Venezuela | 0,272 | 0,292 | 0,279 |
| 6 | Venezuela | 0,323 | 0,344 | 0,333 |
| 4 | Vietnam | 0,070 | 0,080 | 0,080 |
| 5 | Vietnam | 0,094 | 0,105 | 0,107 |
| 6 | Vietnam | 0,131 | 0,143 | 0,146 |

Table S6. Dietary changes shock – Output and Demand simulations (estimations)

|  | **Land supply (estimations)** | | | | | | |
| --- | --- | --- | --- | --- | --- | --- | --- |
|  | **Output** | **Total** | **Household demand** | **Government demand** | **Intermediate demand** | **Investment demand** | **Export demand** |
| **Bovine cattle, sheep and goats, horses** | 368,13 | 742,48 | 57,65 | 1,23 | 301,21 | 14,26 | 368,13 |
|  | *-14,45%* | *-14,50%* | *-20,84%* | *3,53%* | *-13,93%* | *0,87%* | *-14,45%* |
| **Animal products nec** | 588,89 | 1180,38 | 166,74 | 1,19 | 394,4 | 29,16 | 588,89 |
|  | *-13,94%* | *-13,95%* | *-21,18%* | *3,07%* | *-11,48%* | *-0,01%* | *-13,94%* |
| **Raw milk** | 313,36 | 626,72 | 86,69 | 1,18 | 222,36 | 3,12 | 313,36 |
|  | *-15,38%* | *-15,38%* | *-20,51%* | *3,02%* | *-13,48%* | *0,91%* | *-15,38%* |
| **Bovine meat products** | 567,53 | 1140,41 | 357,8 | 0,27 | 213,66 | 1,2 | 567,47 |
|  | *-17,01%* | *-16,99%* | *-22,64%* | *2,46%* | *-5,52%* | *-0,27%* | *-17,01%* |
| **Meat products nec** | 550,61 | 1108,35 | 328,37 | 0,26 | 227,72 | 1,51 | 550,49 |
|  | *-16,55%* | *-16,55%* | *-22,91%* | *2,20%* | *-5,45%* | *-0,35%* | *-16,54%* |
| **Dairy products** | 659,24 | 1323,61 | 390,28 | 0,57 | 271,63 | 2,05 | 659,08 |
|  | *-17,65%* | *-17,65%* | *-23,01%* | *2,58%* | *-8,67%* | *-0,56%* | *-17,65%* |

Numbers in italics represent percentage changes with respect to the values without a shock in the system from Table S8.

Table S7. Dietary changes shock – Output and Demand simulations (0.05)

|  | **Land supply (0.05)** | | | | | | |
| --- | --- | --- | --- | --- | --- | --- | --- |
|  | **Output** | **Total** | **Household demand** | **Government demand** | **Intermediate demand** | **Investment demand** | **Export demand** |
| **Bovine cattle, sheep and goats, horses** | 368,15 | 742,53 | 57,65 | 1,23 | 301,23 | 14,26 | 368,15 |
|  | *-14,44%* | *-14,49%* | *-20,83%* | *3,55%* | *-13,92%* | *0,88%* | *-14,44%* |
| **Animal products nec** | 588,93 | 1180,45 | 166,75 | 1,19 | 394,42 | 29,16 | 588,92 |
|  | *-13,94%* | *-13,94%* | *-21,17%* | *3,09%* | *-11,48%* | *-0,01%* | *-13,94%* |
| **Raw milk** | 313,37 | 626,74 | 86,7 | 1,18 | 222,37 | 3,12 | 313,37 |
|  | *-15,38%* | *-15,38%* | *-20,50%* | *3,03%* | *-13,48%* | *0,91%* | *-15,38%* |
| **Bovine meat products** | 567,55 | 1140,46 | 357,83 | 0,27 | 213,66 | 1,2 | 567,5 |
|  | *-17,00%* | *-16,99%* | *-22,63%* | *2,48%* | *-5,52%* | *-0,27%* | *-17,00%* |
| **Meat products nec** | 550,63 | 1108,39 | 328,39 | 0,26 | 227,72 | 1,51 | 550,51 |
|  | *-16,54%* | *-16,55%* | *-22,91%* | *2,21%* | *-5,46%* | *-0,35%* | *-16,54%* |
| **Dairy products** | 659,26 | 1323,65 | 390,3 | 0,57 | 271,63 | 2,05 | 659,09 |
|  | *-17,65%* | *-17,65%* | *-23,01%* | *2,60%* | *-8,67%* | *-0,57%* | *-17,65%* |

Numbers in italics represent percentage changes with respect to the values without a shock in the system from Table S8.

Table S8. No shock – Output and Demand simulations

|  | **No shock** | | | | | | |
| --- | --- | --- | --- | --- | --- | --- | --- |
|  | **Output** | **Total** | **Household demand** | **Government demand** | **Intermediate demand** | **Investment demand** | **Export demand** |
| **Bovine cattle, sheep and goats, horses** | 430,29 | 868,4 | 72,83 | 1,19 | 349,96 | 14,14 | 430,29 |
|  |  |  |  |  |  |  |  |
| **Animal products nec** | 684,31 | 1371,73 | 211,53 | 1,15 | 445,57 | 29,17 | 684,31 |
|  |  |  |  |  |  |  |  |
| **Raw milk** | 370,31 | 740,62 | 109,05 | 1,15 | 257,01 | 3,09 | 370,31 |
|  |  |  |  |  |  |  |  |
| **Bovine meat products** | 683,83 | 1373,87 | 462,49 | 0,27 | 226,14 | 1,2 | 683,77 |
|  |  |  |  |  |  |  |  |
| **Meat products nec** | 659,78 | 1328,21 | 425,95 | 0,26 | 240,86 | 1,51 | 659,62 |
|  |  |  |  |  |  |  |  |
| **Dairy products** | 800,55 | 1607,33 | 506,93 | 0,56 | 297,42 | 2,06 | 800,35 |
|  |  |  |  |  |  |  |  |

Table S9. Land use change simulations

|  | **Land supply (estimations)** | **Land supply (estimations)** | **No shock** |
| --- | --- | --- | --- |
| **Total managed land** | 6340756,5 | 6345952,5 | 6357971 |
|  | *-0,27%* | *-0,19%* |  |
| **Pasture** | 3194033 | 3196624,75 | 3304029,75 |
|  | *-3,33%* | *-3,25%* |  |
| **Cropland** | 1595359,12 | 1596112,75 | 1505164,12 |
|  | *5,99%* | *6,04%* |  |
| **Forest** | 1551364,38 | 1553214,75 | 1548777,12 |
|  | *0,17%* | *0,29%* |  |
| **Unmanaged Forest** | 2971487,75 | 2967434,5 | 2962369,25 |
|  | *0,31%* | *0,17%* |  |
| **Shrubland** | 743626,81 | 743374,62 | 740570,62 |
|  | *0,41%* | *0,38%* |  |
| **Otherland** | 1921927,5 | 1921929,62 | 1921715,38 |
|  | *0,01%* | *0,01%* |  |
| **Savannah & Grassland** | 1300664,75 | 1299772,38 | 1295837,25 |
|  | *0,37%* | *0,30%* |  |

Numbers in italics represent percentage changes with respect to the values without a shock in the system. Total managed land includes cropland, pasture, and managed forest.

Table S10. Brief overview of available land supply elasticities

| **Authors** | **Area** | **Data** | **Methods** | **Elasticity results** |
| --- | --- | --- | --- | --- |
| Gurgel et al. [2] | Globe | - Tabular panel information - Period from 1990 to 2005 - Land supply indicator: average annual forest converted to forest land - Economic factor: land price changes | -Direct calculation | - Values reported range from 0.12 to 0.60 - Elasticities are aggregated at the country or regional level |
| Barr et al. [3] | US and Brazil | - Tabular panel information - Period from 2003 to 2009 for US and 1997 to 2009 in Brazil - Land supply indicator: observed planted acreage - Economic factor: calculated expected returns | -Direct calculation | - Values reported for US range from 0.005 to 0.028 - Values of elasticity w.r.t. prices are very similar from 0.007 to 0.029 - Values reported for Brazil range from 0.162 to 0.444 (0.003 to 0.122 if including pasture) - Values of elasticity w.r.t. prices are between 0.382 to 0.895 (0.007 to 0.245 if including pasture) |
| Roberts & Schlenker [4] | Globe | - Tabular panel information - Period from 1960 to 2007 - Land supply indicator: area harvested - Economic factor: average futures price | IV with restricted cubic splines | - Values reported ranged from 0.71 to 0.82 (World), -0.095 to 0.289 (US), and 0.261 (Brazil) |
| Liu & Villoria [5] | SSA | - Spatially explicit gridded cross-section at 5 arc-minute resolution - Land supply indicator: harvested area - Economic factor: market accessibility as a proxy for land rents | Spatial Durbin (logit) model | - Majority of grid cells with very inelastic values - Values reported from near zero to up to 2. - At the national level values range between nearly zero to 0.08 |
| Tabeau et al. [6] | Globe | - Tabular (unbalanced) panel information - Various periods tailored for each country - Land supply indicator: Total agricultural area - Economic factor: agricultural value added | -Direct calculation | - Values reported range from 0.019 to 1.357 - Assigned 0.015 for truncated information |
| Villoria & Liu [7] | Americas | - Spatially explicit gridded cross-section at 5 arc-minute resolution - Land supply indicator: cropland share - Economic factor: market accessibility as a proxy for land rents | Fractional response model | - Values reported are from near zero to >.012. |
| Chakravarty & Villoria [8] | US | - Spatially explicit gridded panel at 5 arc-minute resolution. - Period between 2009 to 2017 with additional sub-analyses between 2009 - 2013 and 2013 -2017 - Land supply indicator: cropland share - Economic factor: Land rents accruing to agriculture as main information for elasticities calculation | Fractional response model | - Values reported at the Eco-region range from -0.23 to 0.25. - The differences depend on the region and time period - For the whole US, values are -0.06 (2009-2013), 0.07 (2013-2017), and 0.01 (2009-2017). |


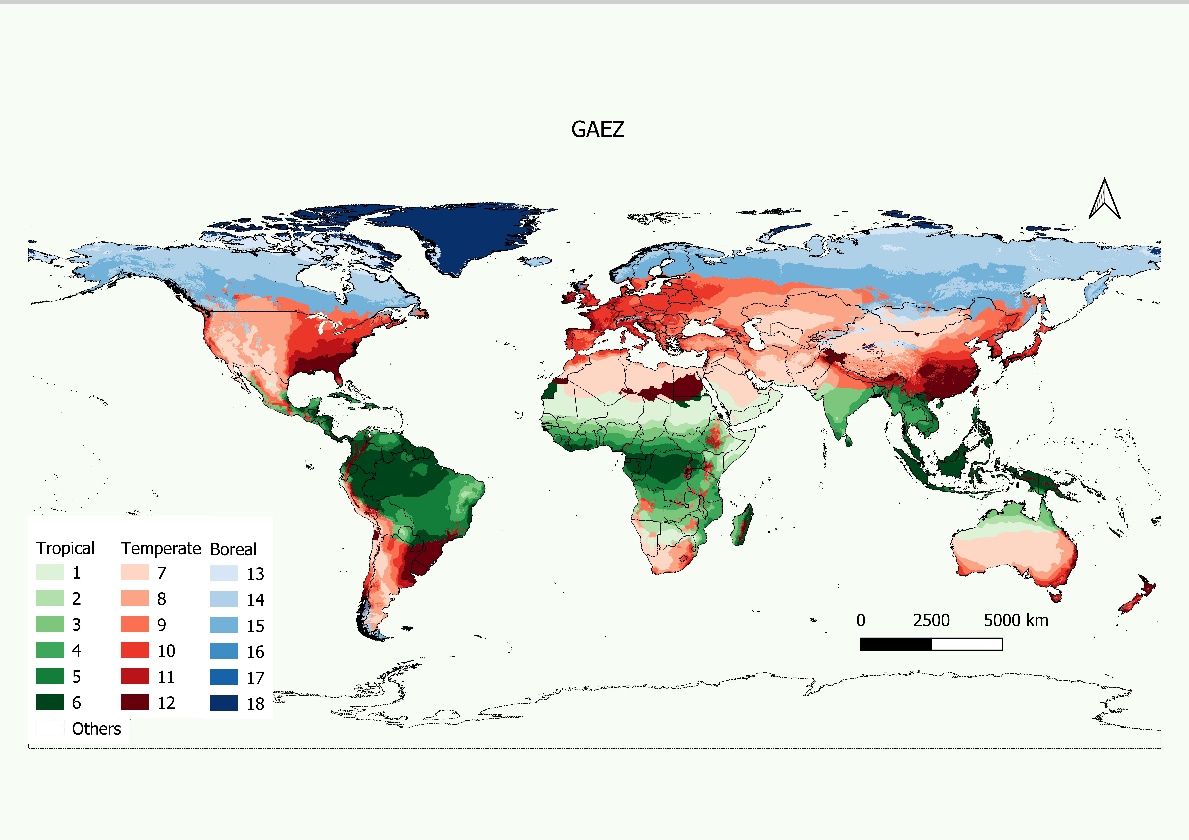


Figure S1. Global AEZ-country observations

The map depicts the intersection of GAEZ and the national boundaries of the world. Adapted from Fischer et al. [9] and Plevin et al. [10].


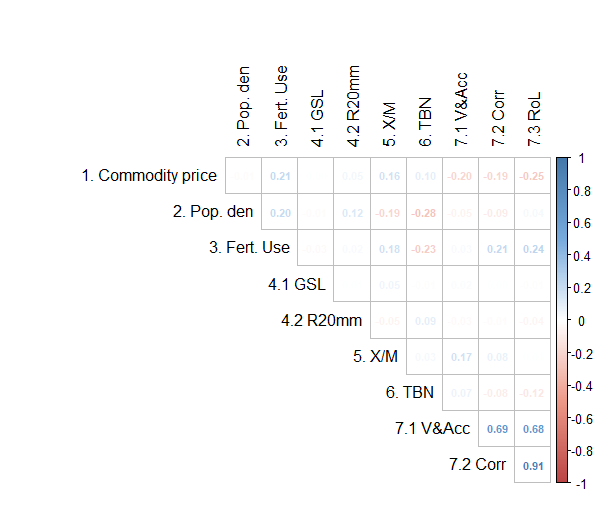


Figure S2. Upper diagonal correlation matrix

Correlation matrix of the covariates used in the econometric analysis. The numbers represent the correlation coefficient, where stronger correlations are depicted with bolder colors.

##
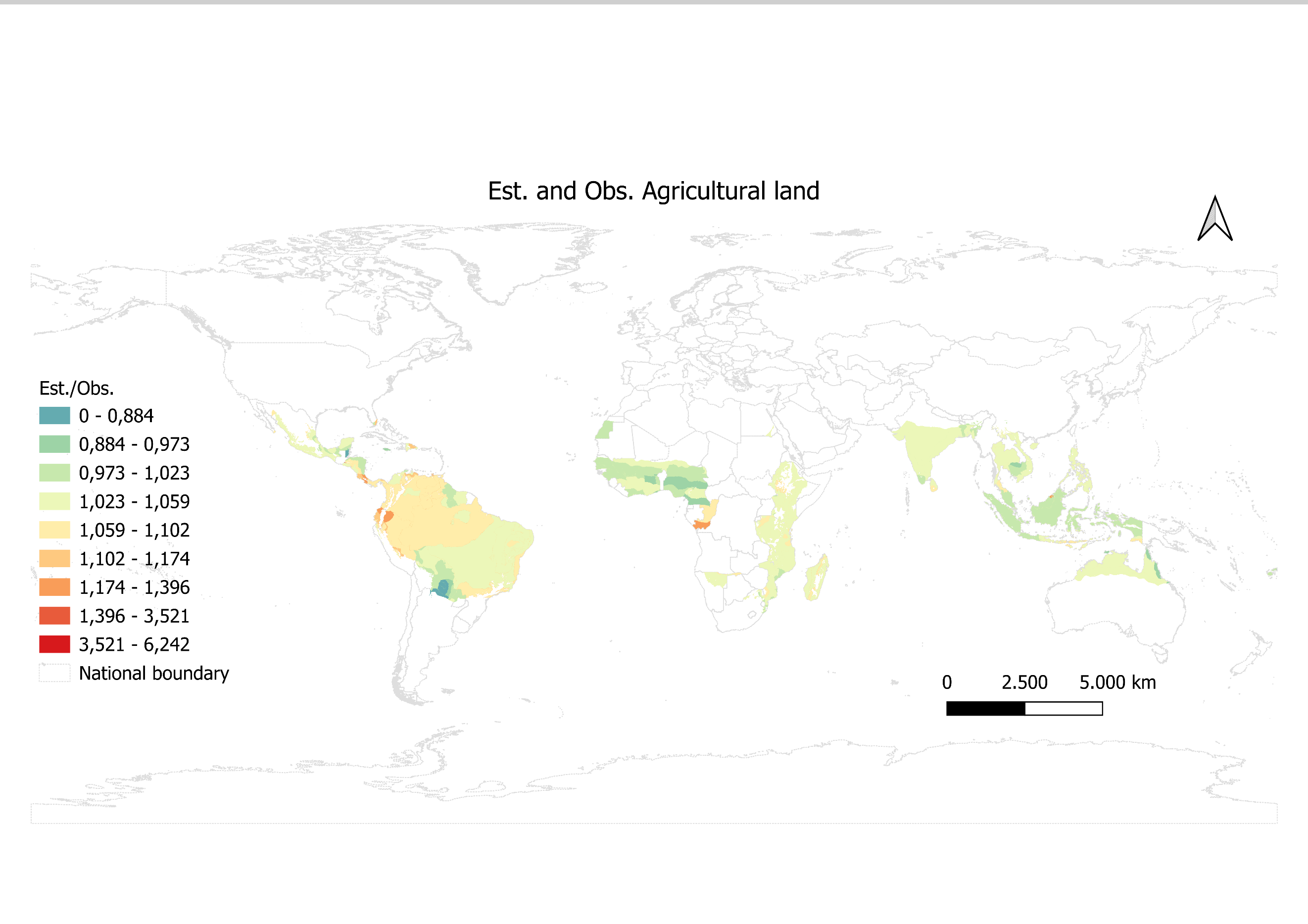


Figure S3. Estimated vs. Observed Agricultural land

The map depicts the ratio of observed and estimated amount of agricultural land for the year 2015. Estimated values are calculated using the observed quantity for the year 2004, our elasticity calculations, and annual percentage price changes reported in FAO’s food price index (<https://www.fao.org/worldfoodsituation/foodpricesindex/en/>).

##
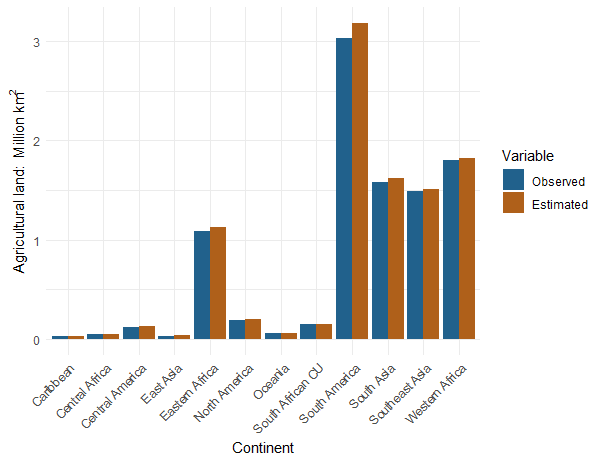


Figure S4. Estimated vs. Observed Agricultural land

Bars depict observed and estimated amount of agricultural land for the year 2015. Estimated values are calculated using the observed quantity for the year 2004, our elasticity calculations, and annual percentage price changes reported in FAO’s food price index (<https://www.fao.org/worldfoodsituation/foodpricesindex/en/>).

References

1. Papke, L. E. & Wooldridge, J. M. Panel data methods for fractional response variables with an application to test pass rates. *Journal of Econometrics* **145,** 121–133 (2008).

2. Gurgel, A., Reilly, J. M. & Paltsev, S. Potential Land Use Implications of a Global Biofuels Industry. *Journal of Agricultural & Food Industrial Organization* **5** (2007).

3. Barr, K. J., Babcock, B. A., Carriquiry, M. A., Nassar, A. M. & Harfuch, L. Agricultural Land Elasticities in the United States and Brazil. *Applied Economic Perspectives and Policy* **33,** 449–462 (2011).

4. Roberts, M. J. & Schlenker, W. Identifying Supply and Demand Elasticities of Agricultural Commodities: Implications for the US Ethanol Mandate. *American Economic Review* **103,** 2265–2295 (2013).

5. Liu, J. & Villoria, N. Profitable cropland available in sub-Saharan Africa, 2016.

6. Tabeau, A., Helming, J. & Philippidis, G. *Land supply elasticities. Overview of available estimates and recommended values for MAGNET* (Publications Office of the European Union, 2017).

7. Villoria, N. B. & Liu, J. Using spatially explicit data to improve our understanding of land supply responses: An application to the cropland effects of global sustainable irrigation in the Americas. *Land Use Policy* **75,** 411–419 (2018).

8. Chakravarty, S. & Villoria, N. B. Estimating the spatially heterogeneous elasticities of land supply to U.S. crop agriculture, 2020.

9. Fischer, G., van Velthuizen, H., Shah, M. & Nachtergaele, F. *Global agro-ecological assessment for agriculture in the 21st century. Methodology and results* (International Institute for Applied Systems Analysis, 2002).

10. Plevin, R. J., Gibbs, H. K., Duffy, J., Yui, S. & Yeh, S. Agro-ecological Zone Emission Factor (AEZ-EF) Model (v47). A model of greenhouse gas emissions from land-use change for use with AEZ-based economic models. GTAP Technical Paper, 2014.

11. Goldman, E., Weisse, M. J., Harris, N. & Schneider, M. Estimating the Role of Seven Commodities in Agriculture-Linked Deforestation. Oil Palm, Soy, Cattle, Wood Fiber, Cocoa, Coffee, and Rubber. Tehnical Note. World Resources Institute, 2020.
